# Animal models for the evaluation of retinal stem cell therapies

**DOI:** 10.1016/j.preteyeres.2025.101356

**Published:** 2025-04-14

**Authors:** Biju B. Thomas, Deepthi S. Rajendran Nair, Mana Rahimian, Amr K. Hassan, Thuy-Linh Tran, Magdalene J. Seiler

**Affiliations:** a Department of Ophthalmology, USC Roski Eye Institute, University of Southern California, Los Angeles, CA, United States; b USC Ginsburg Institute for Biomedical Therapeutics, University of Southern California, Los Angeles, CA, United States; c Department of Ophthalmology, Gavin Herbert Eye Institute, University of California, Irvine, Irvine CA, United States; d Stem Cell Research Center, University of California, Irvine, Irvine, CA, United States; e Department of Physical Medicine and Rehabilitation, University of California, Irvine, Irvine, CA, United States; f Department of Anatomy and Neurobiology, University of California, Irvine, Irvine, CA, United States; g Center for Translational Vision Research, University of California, Irvine, Irvine, CA, United States

**Keywords:** Retinal degeneration, Pluripotent stem cells, Retinal organoids, Retinal disease models, Vision testing

## Abstract

Retinal degeneration (RD) diseases leading to severe vision loss can affect photoreceptors (PRs) that are responsible for phototransduction, or retinal pigmented epithelium (RPE) providing support for PRs. Human pluripotent stem cell (hPSC)-based therapies are a potential approach for restoration of retinal structure in patients with currently incurable RD diseases. Currently, there are two targeted hPSC therapeutics: PR rescue and PR replacement. PR rescue involves the transplantation of RPE or other neural progenitors into the subretinal space to slow down or prevent further RD. RPE transplantation plays a critical role in preserving photoreceptors by providing trophic support and maintaining retinal integrity, particularly in diseases like age-related macular degeneration (AMD). Advances in RPE transplantation methods, such as polarized monolayer cultures and scaffold-based approaches, have shown promise in enhancing graft survival and integration. However, limitations include inconsistent integration, variable neurotrophic factor secretion, and immune rejection risks in non-autologous transplants. In PR replacement, stem cell-derived photoreceptor-like cells or photoreceptor progenitors (PRP) obtained are transplanted into the eye. While PRPs are commonly obtained from retinal organoids (ROs), alternative sources, such as early differentiation stages or direct differentiation protocols, are also utilized to enhance the efficiency and scalability of PRP generation. Challenges include achieving proper integration, forming outer segments, rosette formation, and avoiding immune rejection or tumorigenicity. Various animal models that simulate human RD diseases are being used for establishing surgical feasibility, graft survival and visual functional recovery but fail to replicate clinical immune challenges. Rodent models lack macula-like structures and have limited reliability in detecting subtle functional changes, while larger animal models pose ethical, logistical, and financial challenges. Immunocompromised models have been developed for minimizing xenograft issues. Visual functional testing for efficacy includes optokinetic testing (OKN), electroretinography (ERG), and electrophysiological recordings from the retina and brain. These tests often fail to capture the complexity of human visual recovery, highlighting the need for advanced models and improved functional testing techniques. This review aims to aggregate current knowledge about approaches to stem cell transplantation, requirements of animal models chosen for validating vision benefits of transplantation studies, advantages of using specific disease models and their limitations. While promising strides have been made, addressing these limitations remains essential for translating stem cell-based therapies into clinical success.

## 1. Introduction

Age-related macular degeneration (AMD) and retinitis pigmentosa (RP) are two common outer retinal degenerative (RD) diseases that can lead to severe vision loss. RD diseases target photoreceptors and/or the adjacent RPE. Progressive visual deterioration is caused in retinal degenerative diseases by continuous loss of components of the neural retina, especially the photoreceptors (PR) and Retinal Pigment Epithelium (RPE). RP causes an onset of rod photoreceptor cell loss, which then triggers the late-stage degeneration of cone photoreceptors. Photoreceptor loss eventually causes the death of inner retinal cells, exacerbating vision impairment. RP’s progressive nature and genetic variability pose significant challenges in developing effective treatments. Over 100 associated gene mutations complicate the development of therapies, with oxidative stress and apoptosis playing major roles in photoreceptor degeneration. Gene therapies and iPSC-based regenerative approaches offer promising pathways for personalized treatments (Hamel, 2006; Singh and Nasonkin, 2020; Liu et al., 2022b).

AMD is an RPE degenerative condition that affects the macula - the central region of the retina that is responsible for sharp, photopic vision. AMD is broadly classified into dry and wet forms, with the dry form involving the buildup of drusen (lipid-rich deposits) under the retina, leading to geographic atrophy, and the wet form characterized by abnormal choroidal neovascularization. Both forms significantly impair central vision, affecting daily tasks such as reading and recognizing faces (Khan et al., 2023). As a leading cause of vision loss in older adults, AMD has a profound socioeconomic and emotional impact on patients and their families.

These RD diseases are causes of vision loss which can in turn lead to devastating public health burdens. The diseases not only affect an individual’s quality of life in a negative way, but they also affect the greater society since the burden of central vision loss falls upon the affected individual’s family causing mental and financial stress. Addressing AMD and RP remains a priority in the development of regenerative therapies, particularly stem cell-based approaches (Nowak, 2014; van Lookeren Campagne et al., 2014; Amini et al., 2023).

Two other RD diseases are Diabetic Retinopathy (DR) and Stargardt Disease (STGD). DR is a complication of diabetes mellitus that results in alterations to both neuronal and vascular cells of the retina, with retinal neovascularization being one of the most common complications of DR (Wang et al., 2019). Contrary to the diseases previously mentioned, STGD is the most prevalent cause of inherited blindness in children. While there are varying forms of STGD, STGD1 is the most common which is caused by mutations in the gene ABCA4. Other progressions of the disease manifest with increased macular dysfunction and can be caused by other mutations, such as STGD type 3 being caused by a mutation in the gene encoding ELOVL4. The ABCA4 gene encodes a protein that facilitates the translocation of retinoids within photoreceptor cells. Mutations in ABCA4 impair this function, leading to the accumulation of toxic retinoid byproducts, which subsequently disrupts the visual cycle (Piotter et al., 2021). Mutation in the ELOVL4 gene does not allow for the proper elongation of very long chain fatty acids which play a critical role in PR outer segment lipid bilayers (Kuny et al., 2015). For those suffering from currently incurable RD diseases, stem cell-based therapy is being pursued as a potential approach.

Since the retina is a highly specialized structure, the most promising prospect for retinal repair comes from pluripotent stem cells (PSCs). PSCs such as human embryonic stem cells (hESCs) and induced pluripotent stem cells (iPSCs) can differentiate into a myriad of various cell types. Conversely, autologous multipotent stem cells such as mesenchymal stem cells have drawbacks: they are limited in which cell types they can differentiate into (Liou et al., 2020; Liu et al., 2022b).

In this review, we discuss different approaches to transplantation of stem cell-derived tissues/cells and desirable animal models of retinal degeneration used for testing transplants. Advantages of using specific animal models and their limitations are discussed.

This review focuses on photoreceptor replacement as a regenerative therapy for retinal degenerative diseases. However, given the interconnected roles of retinal pigment epithelium (RPE) and other supporting structures in photoreceptor function, broader search terms encompassing cell therapies for retinal degeneration were used to ensure comprehensive coverage of relevant approaches.

### 1.1. Search strategy and selection criteria

All cited materials were obtained from full-text articles accessed digitally on or before April 10, 2025. Articles were searched on Google Scholar, Scopus, Semantic Scholar and Pubmed using the components “retinal degenerative diseases,” “retinal organoids,” “stem cell transplantation replacing existing photoreceptors,” “retinal stem cells”, “retinal stem cell models” and “retinal degenerative models” in conjunction with a search filter for animal studies. We included peer-reviewed papers discussing the use of stem cells in the treatment and the study of different retinal diseases and excluded papers not published in English, and papers not concerned with retinal stem cells.

### 1.2. Stem cell therapies

For those suffering from currently incurable RD diseases such as AMD and RP, transplantation of stem cell-derived tissue or cells is a potential approach for neuroprotection or cell replacement. PSCs, both ESCs and iPSCs, offer an unlimited source of differentiated RPE and PR material for cell transplantation. hESCs or iPSCs can be differentiated into retinal progenitor cells (RPCs) or photoreceptors and transplanted to replace cells lost in AMD or RP.

**Retinal Progenitor Cells** (RPCs) are mitotically active multipotent stem cells extracted from the developing or adult neural retina (Semo et al., 2016). They combat retinal degenerative diseases in two ways; differentiating into rods and cones (Khalili et al., 2018), and rescuing PRs through neurotrophic factors (Wang et al., 2020).

**Photoreceptor precursors (PRs)**, either derived from early postnatal retina or **pluripotent stem cells (PSCs)** provide significant promise for restoring vision. PSCs, including ESCs and iPSCs, can differentiate into rod and cone photoreceptors, which have been shown to integrate functionally into host retinal tissues in preclinical models. Previous studies using mouse GFP + rod photoreceptor precursors demonstrated successful synaptic connectivity with host retinal cells and improvement in rod-mediated vision in the rd1 mouse model (MacLaren et al., 2006; Lamba et al., 2009; Pearson et al., 2012) and mouse models of cone-rod dystrophy (Santos-Ferreira et al., 2016a). Disease progression had an impact on integration of transplanted cells (Barber et al., 2012; Santos-Ferreira et al., 2016b). Similarly, transplantation of iPSC-derived cone precursors resulted in outer segment formation and functional improvement in cone-degeneration models (Gasparini et al., 2022). However, later studies investigating donor-host material transfer demonstrated that mouse GFP + photoreceptors integrated in the host outer nuclear layer were in reality host cells that had taken up material from donor cells (Pearson et al., 2016; Singh et al., 2016; Ortin-Martinez et al., 2017; Nickerson et al., 2018; Waldron et al., 2018). Yet, a recent study demonstrated that human ESC-derived photoreceptors did not integrate into mouse host retinas (recipients had an intact outer nuclear layer) (Ho et al., 2024).

Recent advancements have focused on improving the survival, orientation, and functional connectivity of transplanted PRs. CRISPR-mediated genetic engineering to remove bipolar cells (Yamasaki et al., 2022) or optogenetic modification (Leger-Charnay et al., 2025), scaffold-based delivery systems (Ballios et al., 2015; Viringipurampeer et al., 2021; Kharbikar et al., 2022), and co-transplantation strategies (Salas et al., 2021; Yang et al., 2021) have enhanced graft integration. For instance, fluorescent reporter PSC lines expressing retinal transcription factors, such as NRL and CRX, have enabled the enrichment of photoreceptor precursors and improved transplant outcomes (Collin et al., 2016; Kaewkhaw et al., 2016; Phillips et al., 2018). Removing the rod-specific NRL transcription factor results in retinal organoids with only cone photoreceptors (Cuevas et al., 2021). Studies using PRPs isolated at the early cone-like stage demonstrated successful host-graft synaptic integration with retinal ganglion cells (Collin et al., 2019; Ribeiro et al., 2021; Zerti et al., 2021).

**PSC-derived retinal organoids** have served as source for transplantation of photoreceptor precursors (Gagliardi et al., 2018; Ribeiro et al., 2021; Gasparini et al., 2022) and retinal organoid sheets (e.g. (McLelland et al., 2018; Tu et al., 2019; Lin et al., 2020; Thomas et al., 2021; Watari et al., 2023; Lin et al., 2024; Sims et al., 2025), (review (Xue et al., 2022; Akiba et al., 2023):) (see section 1.3).

**PSC-Derived Retinal Pigmented Epithelium (RPE)** cells are critical for supporting photoreceptor survival and function, particularly in diseases like AMD, where RPE dysfunction leads to photoreceptor degeneration. Studies in animal models such as RCS rats and pigs have shown that PSC-derived RPE cells integrate into the host RPE layer and slow the progression of photoreceptor loss (Thomas et al., 2016; Sharma et al., 2019; Pishavar et al., 2021). These cells provide essential trophic support, including the secretion of growth factors like PEDF and VEGF, which are necessary for photoreceptor survival and choroidal health.

Recent advancements in transplantation methods have improved RPE graft survival and functionality. For example, RPE cells grown as polarized monolayers on ultrathin biomaterials such as parylene membranes have demonstrated robust integration and functionality in immunodeficient RCS rats (Thomas et al., 2016). Additionally, PSC-derived RPE cells cultured on poly-(lactic-co-glycolic acid) (PLGA) scaffolds have shown promising results in both rat and pig models, requiring fewer cells compared to cell suspensions while maintaining effective integration (Diniz et al., 2013; da Cruz et al., 2018; Sharma et al., 2019; Ye et al., 2020; da Cruz et al., 2024). The use of retinal organoid-derived RPE sheets has also been explored (Liu et al., 2018; Flores-Bellver and Canto-Soler, 2025). These sheets undergo stepwise differentiation into polarized layers, closely mimicking native RPE.

**Mesenchymal stem cells (MSCs)** are non-pluripotent somatic stem cells which can be obtained from bone marrow, umbilical cord (Brown et al., 2022), adipose tissue (Barzelay et al., 2018), and human neural progenitor cells (Jones et al., 2016). While MSCs do not naturally differentiate into photoreceptors, under specific experimental conditions, they can be induced to adopt retinal progenitor-like characteristics. For instance, studies have shown that MSCs can differentiate into retinal progenitor cells when exposed to certain signaling pathway antagonists, suggesting a potential for retinal lineage differentiation (Kicic et al., 2003; Lu et al., 2017; Ding et al., 2019). However, the efficiency and functionality of such differentiation are still under evaluation. In addition to their potential for PR replacement, MSCs have demonstrated the ability to protect degenerating photoreceptors. They can slow the progression of visual loss through the secretion of various immunomodulatory proteins, such as insulin-like growth factor-1 (IGF-1), class II Major Histocompatibility Complex (MHCII) antigens, and Th2-related cytokines especially when employed early (Fan et al., 2020).

### 1.3. Approaches to cell transplantation

There are two main methods of cell transplantation into animal models: injection of single-cells in suspension or implantation of a PSC-derived sheet. There are advantages and disadvantages to each method.

In earlier studies, cell suspension injections of retinal progenitor cells (RPCs) isolated from fetal human eyes (Warfvinge et al., 2011; Klassen, 2016) and human embryonic stem cell-derived retinal pigment epithelium (hESC-RPE) cells (Idelson et al., 2009; Schwartz et al., 2016) were used for transplantation. A key advantage of single-cell transplantation is the ability to target specific cell types, offering flexibility to replace lost retinal cells without the constraints of a pre-formed structure. Additionally, the use of cell surface markers, such as C-Kit+/SSEA4-, allows for enrichment of RPCs and isolation of non-tumorigenic cells from hESC retinal organoids (Zou et al., 2019). The physiological maturation of photoreceptor-like cells derived from human ESCs and iPSCs has been characterized by electrophysiology (Li et al., 2021; Saha et al., 2022), immunohistochemistry (Prameela Bharathan et al., 2021), and transcriptomics (Capowski et al., 2019). There is a critical age for success with isolation of RPCs and PR precursors: cells should still be immature enough to survive the trauma of dissociation and transplantation, but they need to be mature enough to clearly be committed to the retinal lineage and not develop into un-desired cell types (Gasparini et al., 2022). Donor PRs appear to mature faster *in vivo* than in age-matched retinal organoids *in vitro* (Liu et al., 2023c), confirming a previous *in vitro* study that co-culture of retinal organoids with RPE accelerated photoreceptor differentiation (Akhtar et al., 2019).

Major advantages of single-cell transplantation include offering a targeted treatment for loss of certain cell types and avoiding inappropriate synapse formation which can lead to aberrant neural circuitry and functional impairment. Additionally, single-cell transplantation enables control over the purity and quality of cells to minimize the risk of tumorigenesis (review (Xue et al., 2022)). Fluorescent reporter PSC lines expressing key transcription factors in retinal development have been developed to enhance cell enrichment and study cell behavior., e.g., through the replacement of a single neural retina leucine zipper (NRL) allele of the WA09 hESC line with an eGFP or TdTomato reporter gene using CRISPR/Cas9-mediated gene editing, to study and isolate photoreceptor precursors (PRP) (Collin et al., 2016; Kaewkhaw et al., 2016; Phillips et al., 2018). Using this approach, PRPs isolated at the early cone-like stage were shown to integrate successfully into host retinal tissue, with evidence of functional connectivity between donor photoreceptors and second-order neurons, such as retinal ganglion cells (Collin et al., 2019; Ribeiro et al., 2021; Zerti et al., 2021; Gasparini et al., 2022). The enrichment of PRPs through cell surface markers, genetic reporters, and mechanical isolation (Collin et al., 2018) highlights the importance of improving the safety and precision of single-cell transplants. Transplants of iPSC-derived PR retinal organoid cone precursors into adult Cpfl1-mutant mice demonstrated the formation of inner and well-stacked outer segments oriented toward the RPE (Gasparini et al., 2022).

While single-cell transplantation can be effective, the method lacks mechanical stability and control of cell orientation in the graft (Xue et al., 2022). Transplanted cells may form disorganized clusters or rosettes, reducing functional integration. To address these issues, mechanical isolation (Collin et al., 2018) and the use of biomaterial scaffolds to support single-cell grafts have been explored. For instance, the use of polycaprolactone scaffolds has been shown to improve donor cell survival and development in non-human primates (Han et al., 2022). Lee et al. developed a micro molded honeycomb scaffold design to generate a bilayered RPE-photoreceptor cell construct. An optimized photocuring process can make stem cell transplantation more economical and versatile (Lee et al., 2023). Co-transplantation of iPSC-derived RPC and RPE cells in suspension yields better outcomes than transplanting individual cell types in a rat model of retinal degeneration, as evidenced by the earlier showing better conservation of the outer nuclear layer and visual response at 12 weeks (Salas et al., 2021). However, while RPE cells successfully integrated into the host RPE layer, RPCs predominantly remained in the subretinal space, indicating limited integration beyond the site of injection (Salas et al., 2021). In contrast to single-cell transplantation, the implantation of a PSC-derived sheet to the subretinal space provides mechanical support for retinal cells and improves integration due to the complete layered structure of the sheet (Xue et al., 2022). There are two ways to develop PSC-derived sheets for transplantation studies: growing cells on a scaffold made from materials such as poly-(lactic-co-glycolic acid) (PLGA) (Pennington et al., 2015; Koss et al., 2016; Thomas et al., 2016; Gandhi et al., 2020; Ye et al., 2020; Nishida et al., 2021; Rajendran Nair et al., 2021; Yamashita et al., 2023) or developing retinal organoids that undergo stepwise differentiation into layered retinal structures, providing a natural scaffold-free sheet (Lin et al., 2020, 2024; McLelland et al., 2018; Reichman et al., 2017; Thomas et al., 2021; Xue et al., 2021). HiPSC-derived RPE grown on a poly-(lactic-co-glycolic acid)/PLGA scaffold were transplanted into rat and pig models in a study that showed monolayer transplants require ten times fewer cells than cell suspension transplants while maintaining effective integration (Diniz et al., 2013; Sharma et al., 2019). Human RPE has been successfully implanted subretinally into minipigs after being cultured ultrathin nanofibrous carriers with relatively good incorporation into the host retina (Koss et al., 2016; Lytvynchuk et al., 2022). hESC-RPE grown as a polarized monolayer on an ultrathin biomaterial (parylene membrane) showed good survival and functionality in immunodeficient RCS rats (Thomas et al., 2016).

Retinal organoids are a major source of material for transplantation of cell sheets. PSCs are initiated into embryonic bodies and differentiate into retinal layers (Arthur et al., 2022; Xue et al., 2022; Harkin et al., 2024). Subretinal transplantation of ESC and iPSC-derived retinal organoid sheets into retinal degenerative mice and rats led to formation of PRs with outer segments and signs of host-graft synaptic connections (Lin et al., 2020, 2024; Mandai et al., 2017; McLelland et al., 2018; Sims et al., 2025; Tu et al., 2019; Watari et al., 2023). Despite these advantages, PSC-derived sheets pose challenges, including the complexity of surgical implantation and risks of immune response or fibrosis at the graft site. Ensuring the correct orientation and integration of transplanted sheets remains a critical area of investigation.

Finally, mesenchymal stem cells (MSCs) have also been investigated as a supportive strategy rather than direct cell replacement. MSCs, particularly those derived from human umbilical cords, can secrete neuroprotective factors that enhance retinal cell survival (Liang et al., 2022). Filtering MSCs to isolate smaller cells has been shown to improve safety and reduce adverse effects (Liang et al., 2022). However, MSCs do not replace lost retinal neurons directly and are primarily used for their paracrine effects (Mohebichamkhorami et al., 2023).

**Fig. 1.**
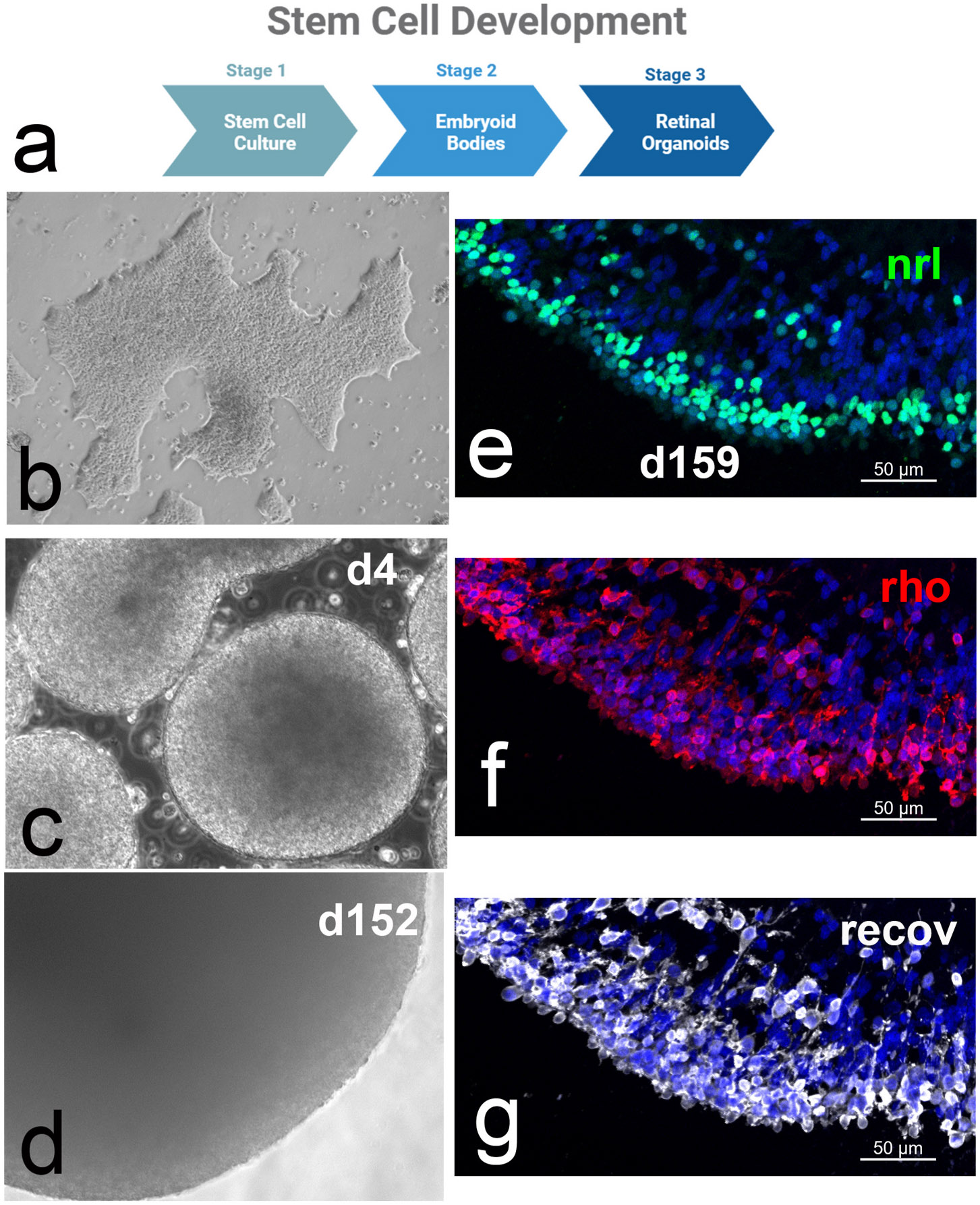
HESC-derived retinal organoids. **a)** Schematic diagram of stages of retinal organoid development; **b)** image of CRX-GFP hESC colony; **c)** embryoid bodies on d4; **d)** retinal organoids on d152; **e-g)** retinal organoids stained for NRL (green), rhodopsin (red), and recoverin (white).

**Fig. 2.**
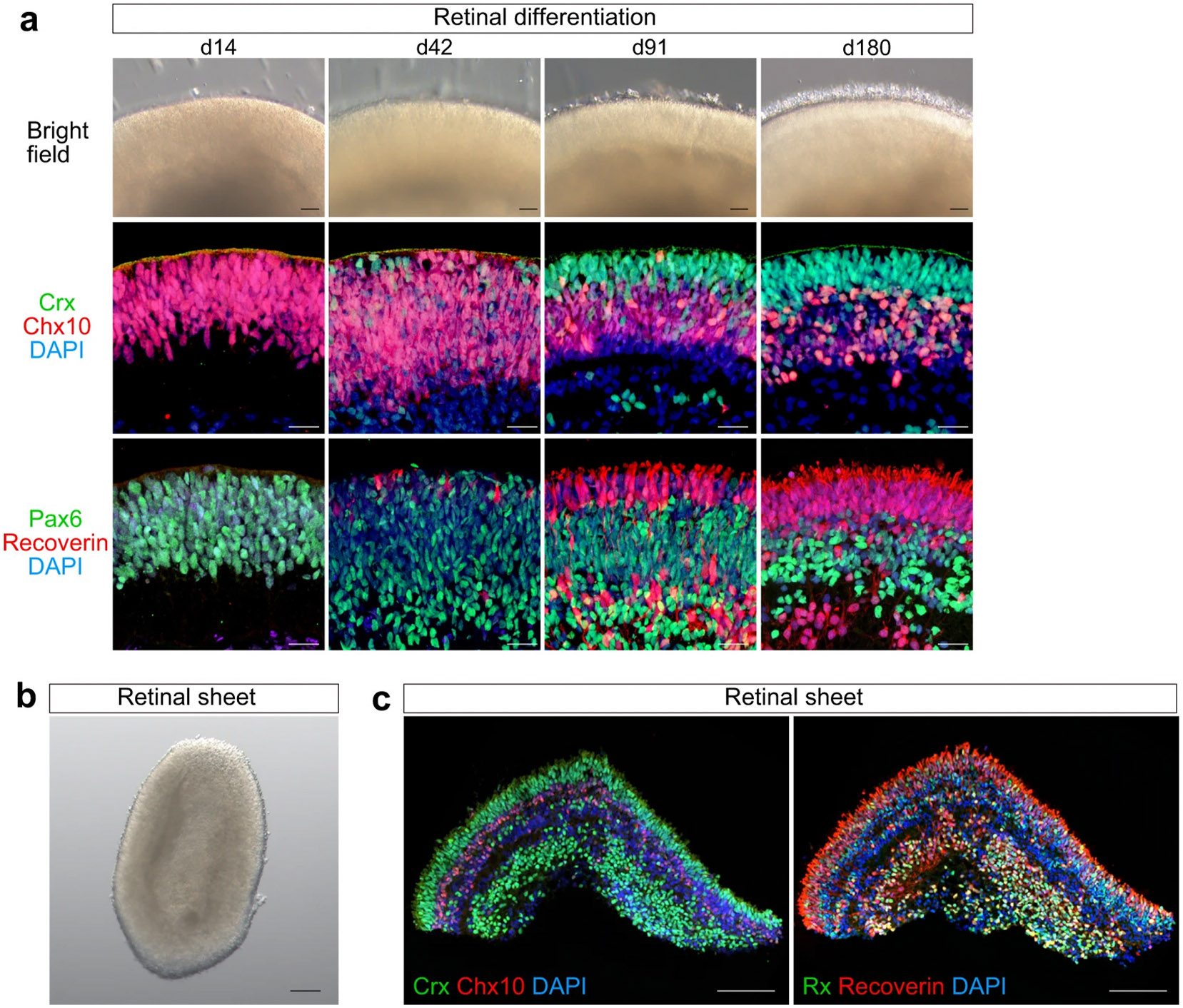
Self-organizing culture of human iPSCs to generate the 3D retina and dissected retinal sheet. Adapted from (Watari et al., 2023), Fig. 1(b-e, f) under a Creative Commons Attribution 4.0 International License (http://creativecommons.org/licenses/by/4.0/). **a)** Bright-field view of iPSC-S17-derived cell aggregate containing retinal tissue on days 14, 42, 91, and 180 (upper). Scale bar in bright-field view: 100 μm. Immunostaining of iPSC-S17-derived retinal tissue on days 14, 42, 91, and 180 (middle and lower). Crx (green) and Chx10 (red) in middle panels. Pax 6 (green) and Recoverin (red) in lower panels. Blue: nuclear staining with DAPI. Scale bar in immunostaining: 20 μm. **b)** Bright-field view of iPSC-S17-derived retinal sheet. Scale bar: 100 μm. **c)** Immunostaining of iPSC-S17-derived retinal sheet on day 87. Crx (green) and Chx10 (red) in left panel. Rx (green) and Recoverin (red) in right panel. Blue: nuclear staining with DAPI. Scale bar: 100 μm.

**Fig. 3.**
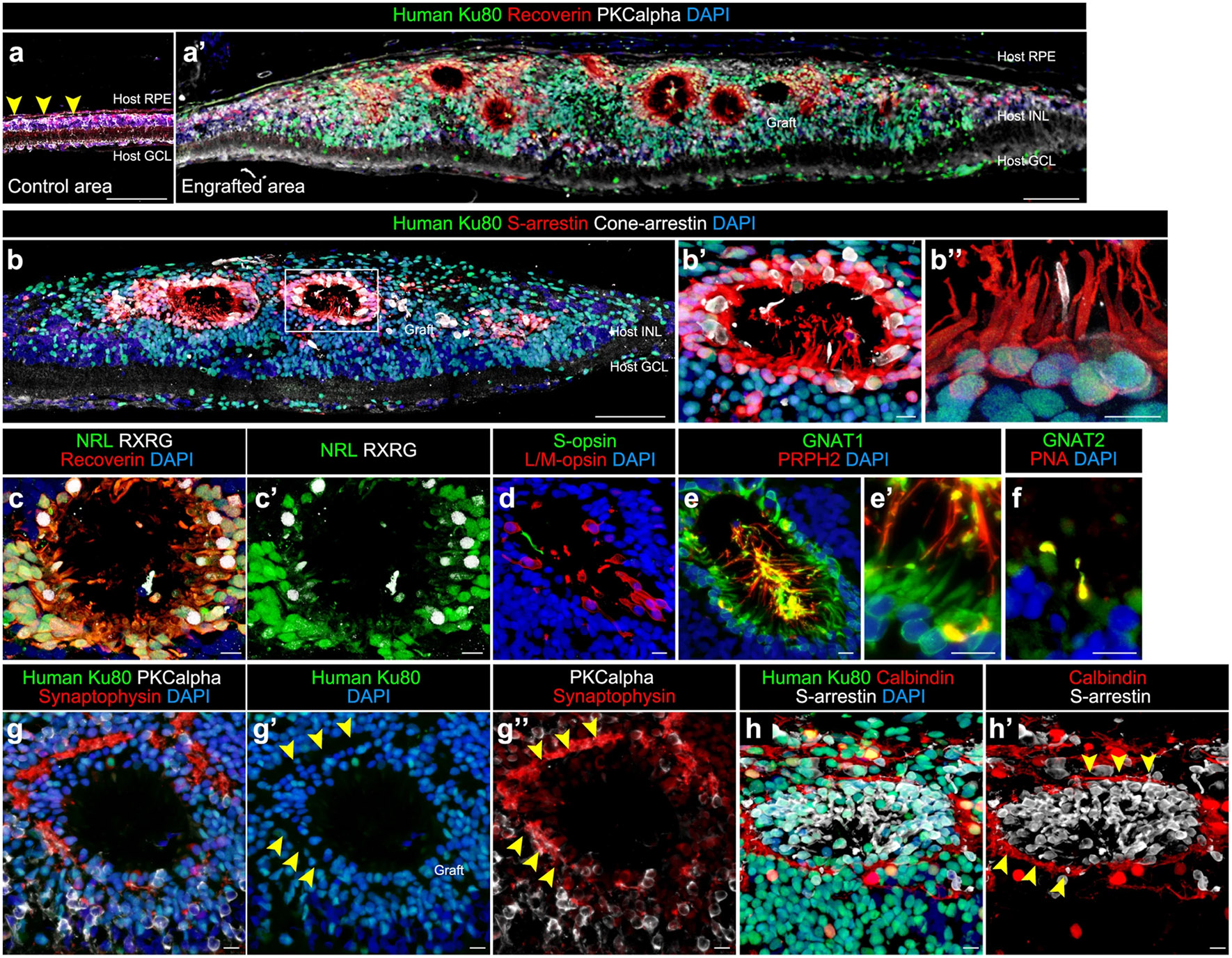
Engraftment and photoreceptor maturation of iPSC-retinal sheets after subretinal transplantation in RD-nude rats. Adapted from (Watari et al., 2023), Fig. 6 (part) under a Creative Commons Attribution 4.0 International License (http://creativecommons.org/licenses/by/4.0/). **a**–**j’** Immunostaining of rat eyes transplanted with iPSC-S17-derived retinal sheets. The retinal sheets were transplanted in the subretinal space of RD-nude rats. The rat retinas were fixed at 263 days after transplantation (341 days after initiation of differentiation). **a**, **a’** Immunostaining for human Ku80 (green), Recoverin (red), and PKCalpha (white). Control non-transplanted area (**a**). Engrafted area (**a’**). Arrowheads in (**a**): human Ku80^−^ , Recoverin^+^ and PKCalpha^+^ rat bipolar cells. **b**–**b”** Immunostaining for human Ku80 (green), S-arrestin (red), and Cone-arrestin (white). Boxed area in (**b**) corresponds to (**b’**). High magnification in (**b’**) and higher magnification in (**b”**). **c**–**h’** Immunostaining for photoreceptor markers. NRL (green), Recoverin (red), and RXRG (white) in (**c**, **c’**). S-opsin (green) and L/M-opsin (red) in (**d**). GNAT1 (green) and PRPH2 (red) in (**e**, **e’**). GNAT2 (green) and PNA (red) in (**f**). Human Ku80 (green), Synaptophysin (red), and PKCalpha (white) in (**g**–**g”**). Arrowheads in (**g’**, **g”**): Synaptophysin-positive neurites in no nuclear space. Human Ku80 (green), Calbindin (red), and S-arrestin (white) in (**h**, **h’**). Arrowheads in (**h’**): Calbindin-positive neurites. DAPI staining (blue) in (**a**, **a’**, **b**–**b”**, **c**, **d**, **e**, **e’**, **f**, **g**, **g’**, **h**). Scale bars: 100 μm in (**a**, **a’**, **b**), 10 μm in (**b’**, **b”**, **c**–**h’**). INL inner nuclear layer, GCL ganglion cell layer.

In summary, single-cell suspension transplants offer flexibility and precision but may suffer from poor structural organization, while PSC-derived sheets provide superior structural integration but require more complex surgical preparation and implantation. The enrichment of cells using surface markers, genetic reporters, and scaffolding technology enhances the survival and functionality of transplanted cells, highlighting the importance of continued optimization of both methods to improve visual restoration outcomes. Fig. 1 demonstrates the timeline of retinal organoid development. Fig. 2 shows the histology of retinal sheets prepared from retinal organoids and Fig. 3 shows the maturation of photoreceptors and bipolar cells in retinal sheet transplants (Watari et al., 2023).

## 2. Animal models used in retinal cell therapy studies

### 2.1. Retinal degeneration models

Various retinal degeneration disease models are currently available for the testing of cell-based therapeutics and understanding mechanisms of visual improvement. These include genetically engineered models that mimic various human retinal diseases. Many models of mice and rats for RP exist, but their use is limited due to the small size of the eye in the rodent. This small size provides limited access to the subretinal space which hinders surgical therapeutic interventions and limits the size of cells that can be injected subretinally or intravitreally. Table 1 summarizes a list of major mouse and rat models used in stem cell therapies.

#### 2.1.1. Mouse models

Mice are advantageous animals to use as models for retinal degeneration research because they can express gene mutations mimicking those in humans. However, the mouse eye is notably smaller than the human eye and has different rod and cone distributions throughout the retina, which may limit the direct translation of findings. One notable example of a model for AMD is the Complement Factor H (CFH) knockout mouse model (Crowley et al., 2023). Another commonly used model is the rd1 mouse, carrying a mutation in the Pde6b gene, is a commonly used model for retinitis pigmentosa (RP) due to its rapid and early photoreceptor degeneration, making it an efficient system for studying therapeutic interventions (Barber et al., 2013; Barnea-Cramer et al., 2016, 2020; MacLaren et al., 2006; Pearson et al., 2016; Ribeiro et al., 2021). Other models, such as *Rhodopsin Pro23His* (P23H) transgenic mice exhibit photoreceptor degeneration in a similar fashion to human RP (Liu et al., 2022c). Mouse models for Stargardt disease harbor a mutated Elovl4 gene, a gene that is involved in the biosynthesis of a membrane-bound protein in photoreceptor cells of the retina. The transgenic *ELOVL4* model is also a good model for AMD (Kuny et al., 2015; Mazzilli et al., 2020). Additionally, the oxygen-induced retinopathy (OIR) mouse model is based on exposing mouse pups to hyperoxia during their phase of retinal vasculature development and is used to investigate DR. It demonstrated a lower area of avascularity and neovascularization after intravitreal injection of bone marrow-derived MSC (Xu et al., 2020).

Therapies for retinal degeneration fall into two distinct approaches: photoreceptor replacement and protection. Replacement strategies aim to restore lost photoreceptors by transplanting photoreceptor precursors or retinal organoids that integrate into the host retina. Studies using rd1 mice have demonstrated successful integration of Nrl-GFP + rod photoreceptor precursors, forming synaptic connections with host retinal cells and improving rod-mediated vision (MacLaren et al., 2006; Pearson et al., 2012). Similarly, transplantation of iPSC-derived cone precursors into mouse models of cone degeneration has shown the formation of inner and outer segments and interactions with host Müller glia, resembling an outer limiting membrane essential for photoreceptor function (Gasparini et al., 2022). While these results are promising, replacement strategies require precise surgical techniques, and long-term cell survival is often limited by immune rejection in immunocompetent models like the rd1 mouse. A study in non-human primates and rd1 mice demonstrated that MHC-mismatched iPSC-retina transplants showed no signs of clinical rejection, although subclinical immune responses were observed. Despite this, these transplants led to significant improvements in visual function, highlighting the potential of allogeneic iPSC-derived therapies to overcome immune barriers (Uyama et al., 2022).

Additionally, efforts to refine retinal organoid-based therapies have been supported by studies comparing mammalian retinal development with iPSC-derived retinal organoids. In this research, developing mouse retinas were used to verify single-cell RNA-sequencing data from iPSC-derived organoids, improving the fidelity of these organoids as a source of replacement photoreceptors. This study could form the basis for developing new therapies for retinal degenerative diseases by enhancing the ability of organoid-derived cells to mimic natural retinal development (Georges et al., 2023). Another study used single cell RNAseq to compare the age-matched development of human retinal organoid transplants in mice with that or retinal organoids *in vitro* and determined that organoid-derived photoreceptors matured faster *in vivo* than *in vitro*; but also identified two migratory cells populations (astrocytes and neural progenitors) *in vivo* that were not found in age-matched retinal organoids *in vitro* (Liu et al., 2023c). While these advancements hold great promise, replacement strategies continue to face challenges, including precise surgical delivery and the impact of immune rejection, particularly in immunocompetent models like the rd1 mouse.

In contrast, protection strategies aim to preserve existing photoreceptors and delay degeneration through the use of neuroprotective factors or cell-based modulation of inflammation. In rd12 mice, transplantation of MSC-derived retinal progenitor cells (RPCs) improved retinal function, reduced inflammation, and enhanced neuroprotection (Brown et al., 2022). Similarly, in NMDA-induced retinal degeneration models, stem cell factor-induced c-kit + cells promoted endogenous repair and improved visual responses (Chen et al., 2022). In models of diabetic retinopathy, such as the OIR mouse, intravitreal injections of bone marrow-derived MSCs restored vascular function and reduced neovascularization (Xu et al., 2020). Protection strategies are less invasive and focus on maintaining retinal structure and function over time; however, they do not replace lost photoreceptors, and their effects may diminish without repeated treatments. CD34^+^ cells are bone marrow stem cells that enhance repair of ischemic tissues in several mouse models including the Streptozotocin (STZ) induced diabetic mouse model (Yazdanyar et al., 2020; Cheung et al., 2021) Protection strategies focus on maintaining retinal structure and function but cannot restore lost photoreceptors, and their effects may diminish without repeated treatments. The rd1 mouse has played a central role in both replacement and protection studies due to its reproducibility and the rapid degeneration of photoreceptors. Its advantages include its cost-effectiveness, availability, and genetic relevance, as its mutation in *Pde6b* mimics aspects of human RP. The rapid degeneration timeline makes it suitable for evaluating therapies in a short time frame. However, its limitations include the severity and speed of degeneration, which do not mimic the slow progression seen in humans. Additionally, the mouse retina’s rod-dominated structure makes it less suitable for studying cone-dominated diseases like AMD. Immune responses can also limit transplantation studies, although immunodeficient strains like rd1/Foxn1^nu can mitigate these issues (Barber et al., 2013; Barnea-Cramer et al., 2016, 2020; Gasparini et al., 2022; MacLaren et al., 2006; Pearson et al., 2012; Ribeiro et al., 2021; Uyama et al., 2022). Despite its limitations, the rd1 mouse, alongside other models such as the P23H transgenic mouse and the OIR model, has significantly advanced our understanding of retinal degeneration and served as a critical platform for testing both replacement and protection therapies. However, to better address the complexities of human retinal degenerative diseases, more sophisticated models are needed. These should aim to replicate the slower, chronic progression of retinal conditions, integrate human-specific mutations, and consider environmental factors that influence disease onset and progression. Incorporating humanized immune systems or engineering more complex genetic backgrounds using CRISPR technology may further enhance their translational relevance.

Additionally, combining small animal models like mice with larger models, such as pigs or non-human primates, may bridge the gap between preclinical and clinical research, enabling more accurate evaluation of therapies. These approaches can help address the remaining challenges of translating findings from mouse models to human patients.

In conclusion, while the rd1 mouse model and other existing models have provided invaluable insights, addressing their limitations and expanding the repertoire of available models will be essential for advancing the development of effective and personalized therapies for retinal degenerative diseases.

#### 2.1.2. Rat models

Rats have larger eyes than mice, making the surgical procedures involved with stem cell transplantation easier. The Royal College of Surgeons (RCS) rats (Zou et al., 2019; Liang et al., 2022), transgenic *Rho-S334ter* rats, and *P23H* rats are commonly used to assess stem cell transplantation therapy for retinal degeneration.

RCS rats have a *MERTK* gene mutation resulting in the RPE losing the ability to phagocytose the photoreceptor outer segments and accumulation of debris in the subretinal space. The resulting progressive photoreceptor degeneration and outer nuclear layer thinning begins around 4 weeks and remains constant until 12 weeks of age as observed in spectral-domain optical coherence tomography (Ryals et al., 2017). The RCS rat model is widely used for studying RPE disease and early-stage interventions using transplants of hESC-derived RPE (Thomas et al., 2016), hiPSC-derived RPE (Shrestha et al., 2020b), and adult RPE stem cell-derived RPE (Farjood et al., 2023). The combined transplantation of hiPSC-derived RPCs and RPE has shown better outer nuclear layer conservation and visual response than isolated RPE or RPC in RCS rats (Salas et al., 2021). However, many studies have demonstrated that RCS rats exhibit protective effects regardless of the type of transplanted cells, whether retinal progenitor cells (RPCs), photoreceptor precursors (PRs), mesenchymal stem cells (MSCs), RPE cells, Müller glia, fibroblasts, or Schwann cells when transplanted at an early age (21-28 d) (Farjood et al., 2023; Jones et al., 2016; Kole et al., 2018; McGill et al., 2007, 2012; Shahin et al., 2023; Surendran et al., 2021; Thomas et al., 2016; Tzameret et al., 2014; Zhang et al., 2021). This suggests that protective effects in RCS rats may result from the immune modulation or trophic factor release common across these transplanted cell types, rather than specific effects attributable to photoreceptor replacement or RPE restoration. Although the RCS rat is useful for studying retinal degenerative mechanisms and cell transplantation, it is not a true model for age-related macular degeneration (AMD). Instead, it may be better described as an AMD-like model, given the overlap in pathology, such as debris accumulation and photoreceptor degeneration.

Transplantation of human cells (xenografts) requires immunosuppressants such as cyclosporine and dexamethasone. Immunosuppressants, however, are less optimal for cell transplantation studies because of inconsistent blood immunosuppression levels, labor-intensive procedures, and additional pain and discomfort to the animals. Immunodeficient RCS rats were created by crossing RCS rats with the athymic nude rat (*Foxn1*^*rnu/rnu*^) to evaluate hESC-RPE xenografts (Thomas et al., 2018). Immunodeficient RCS rats demonstrated transplant longevity of up to 7 months and significant improvement in visual function when transplanted with an iPSC-RPE monolayer (Rajendran Nair et al., 2021), hESC-retinal organoid sheets (Lin et al., 2020), and hESC-retinal organoid and RPE co-transplants (Thomas et al., 2021).

The transgenic *Rho-P23H* and *Rho-S334ter* rats have engineered mutations in the rhodopsin gene (LaVail et al., 2018). The three lines of *P23H* rat have a proline to histidine substitution at codon 23 and the five lines of *Rho-S334ter* rat have a truncation of the last 15 amino acids from the carboxy terminus. These transgenic rat models affect photoreceptor cells and represent a suitable model for inherited retinal degeneration similar to autosomal dominant RP (LaVail et al., 2018). The transgenic lines have been used for transplantation of retinal progenitor sheets (review (Seiler and Aramant, 2012):) and for the assessment of bimodal *in vivo* imaging of hESC photoreceptor precursor cell grafts into the subretinal space (Laver et al., 2015). The immunodeficient *Rho-S334-ter-3* rat (Seiler et al., 2014) has been used in several studies of hESC- and iPSC-derived retinal organoids that demonstrated graft survival, graft-donor cell integration, and recovery of light perception (McLelland et al., 2018; Tu et al., 2019; Watari et al., 2023; Lin et al., 2024).

Rat models depleted of retinal ganglion cells (RGCs) by NMDA and injected with hiPSC-derived Müller glia show improved vision (Eastlake et al., 2019). MSC-derived extracellular vesicles have a positive effect on both the survival and proliferation of PRs and RGCs demonstrated in retinal ischemia rat models (Mathew et al., 2019), glaucoma mouse models (Mead et al., 2018), and optic nerve crush rat models (Cui et al., 2021). The RReSTORe (RGC Repopulation, Stem Cell Transplantation, and Optic Nerve Regeneration) consortium is aiming to initiate collaborations for the repair of the visual pathway in optic neuropathy. It has five main focuses; RGC development and differentiation, transplantation methods and models, RGC survival, maturation, and host interactions, inner retinal wiring, and eye-to-brain connectivity (Soucy et al., 2023).

Recent advancements in retinal degeneration research have expanded beyond photoreceptor replacement and protection to include therapies targeting retinal ganglion cells (RGCs) and broader neuroprotective strategies. For example, studies using hiPSC-derived Müller glia in RGC-depleted rat models have shown improved vision, demonstrating the potential of cell-based approaches for RGC replacement and optic nerve repair (Eastlake et al., 2019). Additionally, mesenchymal stem cell (MSC)-derived extracellular vesicles (EVs) have demonstrated significant neuroprotective effects, promoting the survival and proliferation of both photoreceptors and RGCs in retinal and optic nerve injury models (Mead et al., 2018; Mathew et al., 2019; Cui et al., 2021; Liu et al., 2023b) (review: (An et al., 2025). These findings align with the RReSTORe consortium’s objectives, which aim to integrate advancements in RGC transplantation, survival, and connectivity into broader visual pathway repair strategies.

By combining these approaches, rat models remain instrumental in evaluating therapies targeting multiple cell types, including photoreceptors, RPE, and RGCs, while also addressing neuroprotection and regeneration. However, the inclusion of RGC replacement and EV-based protection alongside retinal degeneration studies emphasizes the need for careful interpretation to ensure alignment with specific research objectives and disease models. Moving forward, rat models will continue to play a central role in testing therapies that bridge foundational research and translational applications, while refining their relevance to human retinal and optic nerve pathologies.

#### 2.1.3. Ground squirrel

A recent rodent model with a cone-dominant retina, the 13-lined ground squirrel, has been used for transplantation of iPSC-derived GRP-expressing photoreceptors (Yu et al., 2024). Transplants were performed 2 weeks after inducing retinal damage (either retinal detachment or ATP injection) and followed for 4 months with Cyclosporine immunosuppression. Transplanted cells migrated into the host retina, and expressed photoreceptor markers (Otx2, Recoverin) but also amacrine and ganglion cell markers.

#### 2.1.4. Rabbit models (Table 2)

Rabbits have large eyes comparable to the size of the human eye, allowing for clinically relevant testing of stem cell therapeutics. However, the rabbit eye contains a merangiotic retina and visual streak in contrast to the holangiotic retina and fovea in primates (Petrus-Reurer et al., 2017).

Stem cell transplantation in rabbits has been studied by inducing characteristics of late-stage dry age-related macular degeneration, known as geographic atrophy (GA), through subretinal injection of sodium iodate (NaIO_3_) (Petrus-Reurer et al., 2017, 2018; Tay et al., 2023). The therapeutic effects of stem cell transplantations in the induced retinal degeneration rabbit model have varied. In one study, subretinal transplants of hESC-RPE in suspension were unable to integrate into induced GA-like rabbit eyes, possibly due to the unique features of the rabbit eye, the nature of the induced degeneration, or immunosuppression protocol (Petrus-Reurer et al., 2017). In a more recent study, hESC-derived PRP were cultured with human retina-specific laminin isoform LN523 before subretinal injection into the induced-degenerative rabbit model and found evidence for integration with host retinal cells (Tay et al., 2023). Beyond evidence of integration, there is a need to understand the mechanism of integration of stem cell transplantation. Using single-cell RNA sequencing, it is possible to observe the single-cell gene expression changes occurring post-transplantation. A trial of transplanted hESC- and iPSC-derived RPE into rabbits analyzed the transcription factors activated post-transplantation to allow for unidirectional maturation of RPE into a structure that would support photoreceptor function (Parikh et al., 2023).

Notably, rabbit models with induced retinal degeneration have increased expression of genes that increase the permeability of the blood-retinal barrier and modulate the immune-privileged status of the eye, making invasive stem cell procedures with this model more prone to graft rejection (Neroeva et al., 2022). Rabbits are large-eyed animal models that can be easily housed and allow for the testing of clinically relevant surgery and imaging techniques.

#### 2.1.5. Canine and feline models (Table 2)

Several canine (Bortolini et al., 2023; Pasmanter et al., 2021; Ripolles-Garcia et al., 2022b,c) and feline (Aplin et al., 2016; Minella et al., 2018; Occelli et al., 2016; Singh et al., 2019) models of inherited RP have been identified but there are several major limitations in these species including limited genetic information, the presence of major and minor histocompatibility differences in using cell-based therapies, the societal concern over experimentation in them, and the limited access to retinal progenitor and stem cells in these species. Cat models have been used for the transplantation of fetal retinal sheet allografts (Seiler et al., 2009) and transplantation of hESC-derived organoids (Singh et al., 2019; Occelli et al., 2021). Photoreceptor precursors derived from hESC-retinal organoids expressing CRX-TdTomato were injected into the subretinal space of rcd1/PDE6B dogs and showed synaptic maturation at 3–5 months post-transplantation (Ripolles-Garcia et al., 2022b). Canine iPSCs have been developed and identified a unique intermediate pluripotent state distinct from mouse and human iPSCs. While these ciPSCs have not yet been used in transplantation studies, they offer a promising tool for species-specific disease modeling and future regenerative therapies in dogs. (Menon et al., 2021). Bone marrow stem cells transduced with a lysosomal tripeptidyl peptidase-1 (TPP1) expression construct prevented retinal degeneration in a canine model with a TPP1 null mutation, a model of neuronal ceroid lipofuscinosis (Tracy et al., 2016).

#### 2.1.6. Pig models (Table 2)

A pig’s eye is ideal given its similarity to the human eye in size and structure, especially with the rod photoreceptor dominant periphery and the cone dominant center. In addition, subretinal hESC- and hiPSC-derived RPE have successfully been transplanted into pig models (Koss et al., 2016; Lytvynchuk et al., 2022). However, the large body size of the pig limits its utility and widespread use. Laser-induced RPE ablation (Sharma et al., 2019; Barone et al., 2023) and NaIO_3_ subretinal injection (Duarri et al., 2021) were used in pigs to induce retinal degeneration. Transgenic pig models of autosomal dominant RP were created by somatic cell nuclear transfer (SCNT) using bacteriophage EMBL3 that expresses the Pro23His (P23H) Rhodopsin (RHO) mutation (Scott et al., 2017). There is also a transgenic pig model of cone dystrophy (Kostic et al., 2013), Usher’s syndrome (Grotz et al., 2022) and of AMD (ELOVL4 transgenic pigs) (Sommer et al., 2011a).

Transplantation of clinical grade iPSC-derived RPE on a biodegradable scaffold (Sharma et al., 2019) and as single cell suspensions (Duarri et al., 2021) demonstrated findings of integration including retinal phagocytosis by the transplanted RPE cells. Adjuvant material can also help with proper graft delivery and survival, as was demonstrated by a pig’s model of RP that had subretinal cell graft accompanied by polycaprolactone (PCL) for better survival (Thompson et al., 2019).

#### 2.1.7. Non-human primate (NHP) models (Table 2)

**Fig. 4.**
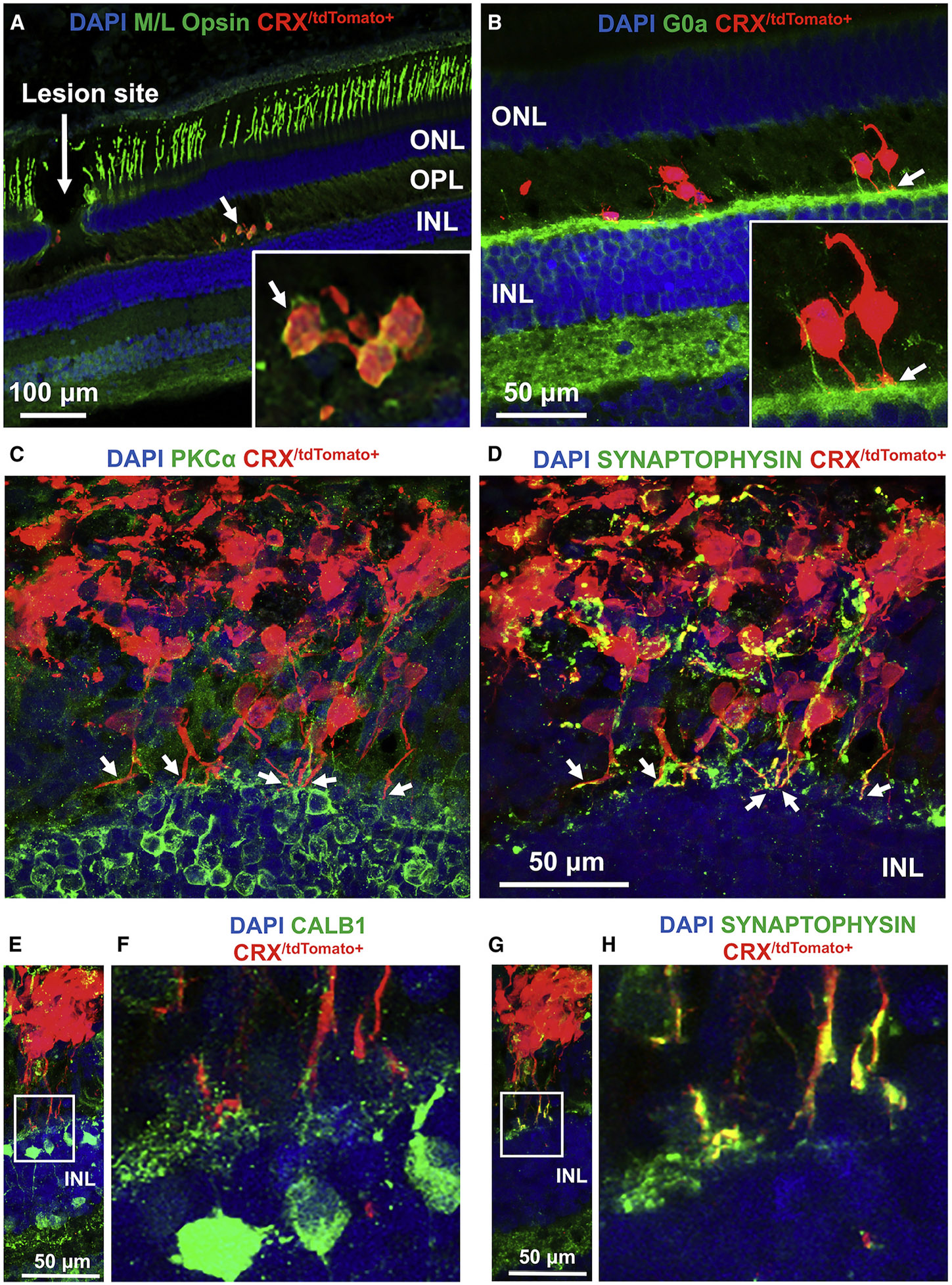
Immunohistochemical Examination of Photoreceptor Precursor Integration and Differentiation. Adapted from (Aboualizadeh et al., 2020), Fig. 6, under a Creative Commons Attribution 4.0 International License (http://creativecommons.org/licenses/by/4.0/). **(A)** Some migratory transplanted photoreceptor precursors matured *in vivo* to express M/L opsin. **(B)** Neurites from transplanted cells were in contact with host second-order neurons, including G0α+ ON bipolar cell dendrites. **(C** and **D)** Some donor photoreceptor precursors extended axons toward host bipolar cells (PKCα+, **C)** and expressed the presynaptic protein marker (synaptophysin) **(D)**. While expression of synaptophysin in transplanted cells was disorganized in cells away from the host OPL, processes close to the host INL had pronounced expression of synaptophysin in putative axonal terminals.**(E**–**H)** Some photoreceptor precursors contacted host second-order neurons, including CALB1+ horizontal and bipolar cells **(E** and **F)** and expressed synaptophysin **(G** and **H)**. The white squares in **(E)** and **(G)** are shown magnified in **(F)** and **(H)**, respectively.

There are many NHP models of retinal degeneration (review (Picaud et al., 2019; Ail et al., 2023); details are outside the scope of this article). Monkey models work well because the monkey has a fovea and human-like visual perception with a similar immune system to man (Aboualizadeh et al., 2020). This foveal similarity is most vital when examining cone-predominant retinal degenerations. In fact, Moshiri and colleagues (Moshiri et al., 2019) have identified four NHP models for cone disorders, which may help in the search for achromatopsia gene replacement, as well as optimization of gene editing in the macula and cone cell replacement in general. An NHP laser damage model was used for the transplantation of ESC- and hiPSC-derived retinal sheets; the development of transplant photoreceptors was documented 4 months after transplantation (Shirai et al., 2016; Tu et al., 2019). The same research group also documented that MHC-mismatched ESC-retinal transplants showed graft development without lymphocyte infiltration, although subclinical signs of rejection were observed (Uyama et al., 2022); and reported about adaptive topics SD-OCT analysis (AO-OCT) of NHP primates and of one RP patient with a retinal organoid transplant (Ishikura et al., 2024). They also optimized laser ablation for RPE damage and subsequent transplantation of hiPSC-RPE strips (Kajita et al., 2024). Another group transplanted *CRX-TdTomato* expressing photoreceptor precursors (derived from retinal organoids) to normal and laser-injured retinas of non-human primates (Aboualizadeh et al., 2020). Subretinal transplants to non-lesioned retina remained separated from the host retina, whereas in laser-injured retina, photoreceptor precursors migrated towards the host IPL (Fig. 4). Table 2 summarizes the major differences between different animal models.

### 2.2. Further classification of animal models based on testing safety and efficacy, and surgical feasibility

Studies using animal models for testing stem cell transplantation studies of the retina can be broadly classified into 1. Safety and efficacy studies and 2. Surgical feasibility studies.

#### 2.2.1. Safety and efficacy studies using animal models

Animal models used for safety and efficacy studies are generally required in large numbers (N). Such a high ‘N’ is required for obtaining meaningful statistical analysis. Justification of the animal numbers based on statistical power analysis can be a requirement for funding agencies and IND filing. Since the majority of the safety studies include long-term validation of transplant survival and the absence of tumorigenesis and xenograft issues, such studies are time-consuming and hence generally expensive. Several CROs are currently available to conduct GLP-level preclinical animal experiments that are often required for IND filing. Due to cost-effectiveness and the availability of suitable disease models, the majority of such GLP studies are conducted using small animal models (rats and mice) (Lin et al., 2017). In certain cases, large animal models such as pigs (Del Priore et al., 1995; Petters et al., 1997; Ghosh et al., 2004; Klassen et al., 2012; Duarri et al., 2021), dogs (Petersen-Jones et al., 1999; Acland et al., 2001; Zhang et al., 2002; Pichard et al., 2016; Guziewicz et al., 2018; Makelainen et al., 2019; Winkler et al., 2020) and even non-human primates (Shirai et al., 2016; Moshiri et al., 2019; Peterson et al., 2019; Tu et al., 2019; Winkler et al., 2020; Gong et al., 2023) are also used (Negi et al., 2023) (see Table 2).

Two major limitations in using animal models for studying cell replacement therapies include lack of adequate disease manifestation and xenograft rejection issues. The former limitation cannot be totally circumvented considering the lifespan of rats and mice disease models. In humans, progressive degenerative diseases of the retina including RP and AMD peak over a period of several decades. Compared to disease manifestation issues, immunological problems associated to xenograft are comparatively easy to overcome by using suitable immunosuppressant regimes (Singhal et al., 2008; Pearl et al., 2011; Rong et al., 2014; Xian and Huang, 2015; Cooper et al., 2016; Koss et al., 2016; Fujii et al., 2020; Liu et al., 2021) (review (Diehl et al., 2016)) or by using immunodeficient disease models (Diniz et al., 2013; Kanemura et al., 2014; Seiler et al., 2014; Shirai et al., 2016; Zhu et al., 2017; Iraha et al., 2018; Thomas et al., 2018; Kim et al., 2021; Zhang et al., 2021).

Use of immune-suppressants to minimize graft rejection (Table 3): Several previous cell therapy preclinical studies were conducted in animal models. In these studies, xenograft issues raised concerns about transplant survival and innate tissue reactions that could adversely affect the therapies’ beneficial effects. To minimize such issues, immunosuppressants were used (Singhal et al., 2008; Pearl et al., 2011; Rong et al., 2014; Xian and Huang, 2015; Cooper et al., 2016; Koss et al., 2016; Fujii et al., 2020; Sims et al., 2025). For rat studies, prednisolone was a generally accepted immunosuppressant applied in drinking water (Singhal et al., 2008). However, long-term use of such immunosuppressants causes adverse effects in animals which is less desirable for obtaining consistent data outcome (Cooper et al., 2016). Further, the use of immunosuppressants was found to affect functional assays and animal health (Cooper et al., 2016). In large animal models, for ocular transplantation studies, intravitreal delivery of slow-releasing Ozurdex was used as an immunosuppressant drug (Xian and Huang, 2015; Koss et al., 2016). The use of Ozurdex is appealing since the same drug has been used for human patients to minimize immune reactions following RPE transplantation (Koss et al., 2016).

Use of immunodeficient animal models (Table 4): To overcome several issues associated with oral administration and or injection of immunosuppressants, immunodeficient animal models are preferred (Anderson et al., 2011). In earlier studies, immunodeficient mice and rats (SCID) were used to avoid xenograft rejection (Kanemura et al., 2014; Kim et al., 2021). More recently, immunodeficient rodent disease models that mimic human RD conditions were generated mostly by cross-breeding. This includes models for RP disease (immunodeficient *Rho-S334ter* line 3 rats (Seiler et al., 2014; Shirai et al., 2016; Seiler et al., 2017; Lin et al., 2018; McLelland et al., 2018; Tu et al., 2019; Uyama et al., 2022) and AMD (immunodeficient *RCS* rats) (Seiler et al., 2014; Thomas et al., 2018), or mouse models with deletion of Interleucin-2γ-receptor (IL2Rγ) expression (Zhu et al., 2017). Studies conducted using the above animal models showed better graft survival and visual functional outcomes (Zhang et al., 2021).

#### 2.2.2. Animal models used for testing surgical feasibility and surgical tools (Table 5)

Different types of surgical tools and techniques have been developed and tested in animal models for subretinal delivery of RPC as sheets and RPE as a polarized monolayer (Hu et al., 2012; Al-Nawaiseh et al., 2016; Koss et al., 2016; Li et al., 2022; Martinez Camarillo et al., 2022). Animal models can have normal (non-disease) or diseased eyes (da Cruz et al., 2007; Suzuki et al., 2007; Al-Nawaiseh et al., 2016; Thomas et al., 2016; Thomas et al., 2018; Duarri et al., 2021).

These studies are aimed at demonstrating the surgical feasibility of the transplantation procedure. Another important aspect that needs to be considered here is that transplantation of an RPE or retinal organoid sheet requires highly trained surgical skills. The above experiments mostly involve two phases 1). Development of a tool and 2). Testing the tool in a suitable animal model with eye size comparable to that of humans.

## 3. Validating the functional benefits of cell replacement therapies in animal models

Functional assessment of visual function can be performed by ERG, retinal multielectrode arrays (MEAs), brain recordings or behavioral visual acuity tests which demonstrate visual responses mediated through the central neural circuitry. These are particularly useful in assessing rodent models of retinal degeneration (McLelland et al., 2018; Lin et al., 2020; Thomas et al., 2021; Yang et al., 2021; Tay et al., 2023). Vision testing in animals can be broadly classified into two types-those based on electrophysiology and those based on behavioral testing (see Table 6, Table 7).

### 3.1. Visual behavioral testing in rodents

Unlike in humans, validation of vision in rodents based on behavioral assessment is more challenging. Two major types of vision testing are employed in rodents. 1. Tests that require prior training and 2. Involuntary head-tracking based on optokinetic nystagmus.

#### 3.1.1. Tests that require prior training (Fig. 5)

This includes the use of special equipment such as a water maze and visual discrimination apparatus, both require extensive training for the animals before the actual testing. These trainings ensure that the results accurately reflect their visual capabilities rather than other factors such as stress or unfamiliarity with the testing environment.

##### 3.1.1.1. The Morris water maze.

**Fig. 5.**
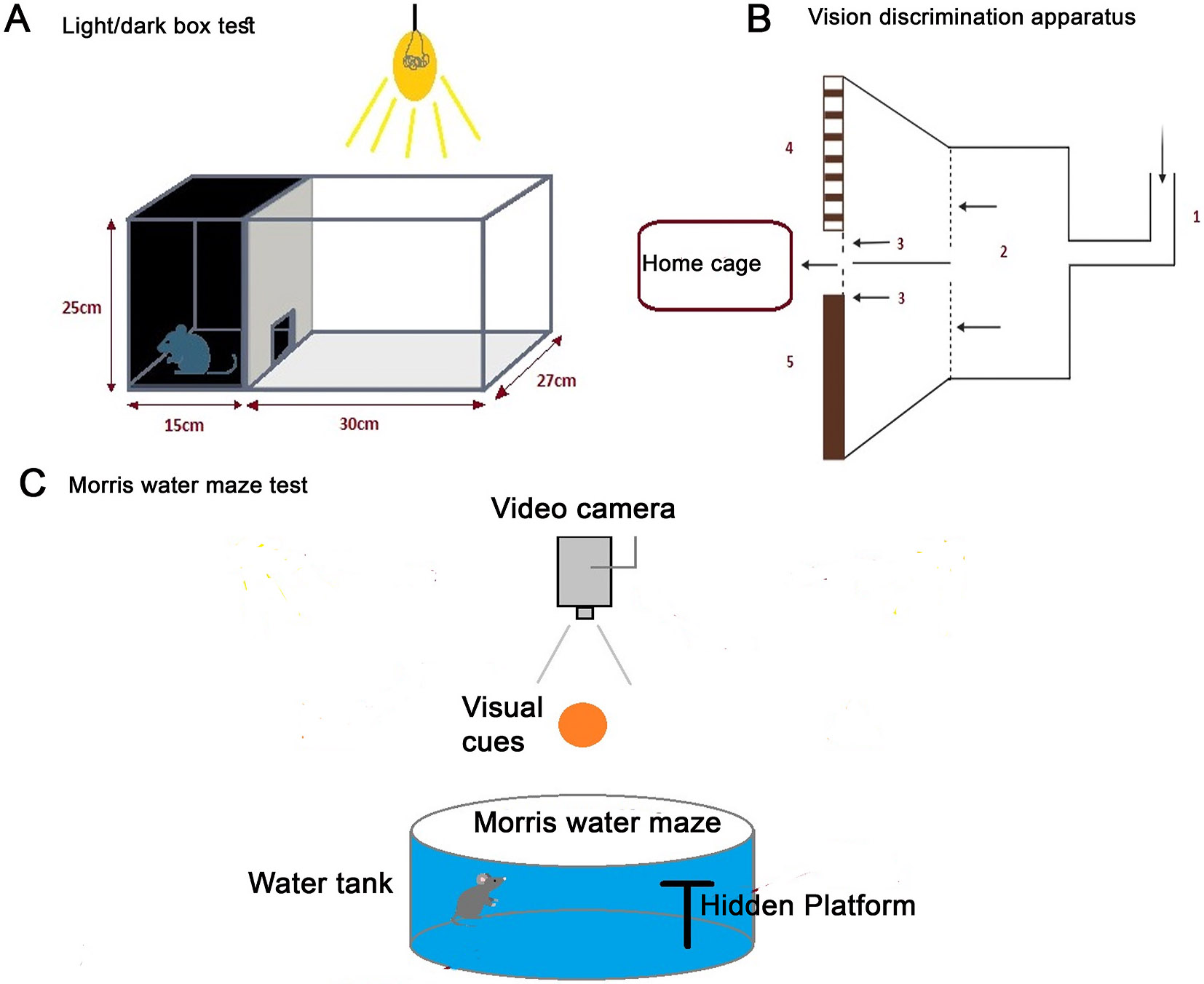
Instruments used for training rodents for vision testing. A. Light dark box, apparatus. Dark/Light Box for Visual Activity Testing: The apparatus capitalizes on rodents’ natural aversion to brightly lit spaces and their exploratory behavior when faced with mild stressors, such as a novel environment and exposure to light. It consists of a small dark ‘safe’ compartment (one-third) and a larger illuminated ‘aversive’ compartment (two-thirds), enabling the assessment of visual function and anxiety-related responses in rodents."-B. Visual discrimination box, schematic drawing. Rats are introduced through a bend tube (1) that leads to the introduction chamber (2) from where the rats can move into one of the two escape alleys (3) by passing the transparent swing doors (dotted lines). At the far end of the escape alleys, a second set of swing doors are present which open into the home cage. The rats are trained to find the unlocked exit door based on the ‘positive’ visual stimulus displayed on one of the computer monitors (4), whereas a ‘negative’ visual cue is displayed on the other side B. Vision discrimination apparatus The open-field test box consisted of a dark compartment (one third of the floor area) and a larger illuminated compartment (two thirds). A small opening located at floor level in the center of the dividing wall allowed mice to freely move between the lit and dark chambers. C. Morris Water Maze. This set up-assesses visual function and spatial learning in rodents by requiring them to locate a hidden platform using visual cues around the pool. Impairments in vision are indicated by increased time or difficulty in finding the platform.

The Morris Water Maze is widely used for vision testing in laboratory rats (Prusky et al., 2000; D’Hooge and De Deyn, 2001; Vorhees and Williams, 2006) and mice (Prusky et al., 2000; Vorhees and Williams, 2006; Wong and Brown, 2006; Brown and Wong, 2007). As a widely used tool in behavioral neuroscience, the Morris Water Maze primarily focuses on assessing spatial learning and memory. However, it can also be utilized in other studies, such as visual testing. During these tests, rodents are trained to find a hidden escape platform based on visual cues they learn during training. The platform is submerged just below the water’s surface, making it crucial to train the rodents under wet conditions to ensure they can navigate effectively. The Morris Water Maze has various adaptations that allow researchers to study different visual conditions; for example, it can be used to assess the visual capabilities of each eye independently. However, this method requires animals to be trained in a water tank, which can be stressful for the subjects (Harrison et al., 2009) (Fig. 5).

##### 3.1.1.2. Visual discrimination apparatus.

In contrast to water maze testing, a visual discrimination apparatus avoids forcing animals to swim by offering a dry and controlled environment for visual cognition and perception testing. For instance, Prusky et al. developed a virtual optometer system that tracks head movements in response to visual patterns, allowing researchers to monitor visual acuity without the stress associated with water-based tasks (Prusky et al., 2000). Similarly, Bussey et al. introduced a touchscreen-based VDA, enabling rodents to perform visual discrimination tasks in a dry environment (Bussey et al., 2001). This method involved training the animals to distinguish between different visual patterns on a touchscreen, thus eliminating the complications related to water immersion. Hannesson et al., 2004 also used a dry maze to study how damage to the hippocampus affects a rodent’s ability to recognize and remember visual patterns (Hannesson et al., 2004). Matsuo et al. used an operant conditioning test to reward rats for recognizing a light stimulus (Matsuo et al., 2019).

#### 3.1.2. Tests that do not require prior training (Fig. 6)

##### 3.1.2.1. Optokinetic test.

Optokinetic (OKN) head-tracking is an involuntary response mediated by subcortical centers, including the superior colliculus (SC); and is an assessor of visual function in rodents. Headtracking consists of compensatory eye movement in the direction of a stimulus movement (usually high-contrast alternating stripes with varying width, i.e., grating frequency). The tested animals turn their head in an attempt to track moving stimuli, followed by turning the head back and restarting the tracking process.

Higher mammals with considerable overlap of their visual field and good binocular vision have similar responses to stimulation in both directions of rotation during monocular optokinetic stimulation. The visual cortex is responsible for this symmetry between nasotemporal and temporonasal stimulation; hence it follows that humans with asymmetry in optokinetic response develop so in response to immature cortical systems, as is seen in infants (review in (Robinson, 2022).

To measure visual acuity in rodents, the total time spent tracking the movements is scored at different spatial frequencies (Coffey et al., 2002). The apparatus has been widely used by investigators for testing transplant effects in rodent disease models. Eyes that undergo cell therapies elicit higher levels of OKN responses compared to controls (Tsai et al., 2015; Davis et al., 2017; McLelland et al., 2018; Lin et al., 2020; Liu et al., 2020; Mitrousis et al., 2020; Thomas et al., 2021). Devices that measure the optokinetic response in both eyes simultaneously (Coffey et al., 2002) as well as in monocular eyes (Thomas et al., 2004b; Ahmed et al., 2022) have been described, and while the temporal-nasal direction was comparable in both methods, the nasotemporal direction was significantly less sensitive in the monocular eyes approach. Since rodents do not have much overlap in visual fields between both eyes, responses between both eyes can be distinguished by changing the direction of the stimulus. A major limitation in rodent vision testing is the inconsistency and lack of reliability, particularly in detecting subtle differences in visual function. These tests often fail to fully capture the complexity of human visual perception and functional recovery, highlighting the need for developing advanced macula-like models and refining functional assessment techniques to improve preclinical validation.

##### 3.1.2.2. Light/dark box testing.

Rodents naturally prefer darkness during the day. The light-dark box test measures the preference of the tested rat/mouse between a lighted box and a dark box (Pearson et al., 2012; Matsuo et al., 2019; Ribeiro et al., 2021). It can clearly show the difference between rodents with retinal degeneration and wild-type animals with normal vision.

Incorporating the light-dark box test as a behavioral measure of visual restoration could provide additional insights into the effectiveness of such transplantation therapies. This test evaluates light preference behavior in transplanted animals, offering a quantitative approach to compare outcomes between degenerated, transplanted, and wild-type models. Behavioral assessments, combined with physiological evaluations like ERG and histological analyses, strengthen the evidence for functional recovery following retinal cell transplantation. For instance, PRP transplantation has been shown to enhance light perception in NOD. SCID-rd1 mice, supported by positive electroretinography (ERG) signals indicating functional photoreceptor activity in the outer nuclear layer (Liu et al., 2023a). Similarly, RPE transplantation in RCS rats demonstrated morphological and functional protection, with survival and integration of transplanted cells (Surendran et al., 2021).

### 3.2. Visual behavioral testing in primates (Table 7)

Unlike in rodents, visual behavioral testing in primates is more advanced and reliable due to the ability of these animals to detect complex visual patterns like humans (Bruce et al., 1981; Orban, 2011).

For instance, in face perception studies, primates like rhesus monkeys are trained to recognize human faces to understand the neural mechanisms underlying face recognition in both humans and primates. Since primates have more developed visual and cognitive systems, they can effectively perform these recognition tasks. However, it is very challenging for rodents to achieve the same level of recognition. This helps us understand why primates are better suited for processing complex visual stimuli (Tsao et al., 2006; Gothard et al., 2007).

The training phase in primates is predominantly based on positive reinforcement, such as food rewards or social interaction, which enhances the effectiveness of the study, improves ethical considerations, and reduces the stress experienced by the primates. For example, in the study by Gaffan et al., 1989, macaques were trained to perform visual discrimination tasks using a visual secondary reinforcer, with a preferred treat provided only after successfully solving the problem, making the process more humane and efficient. Similarly, baboons were tested in a social group setting and were rewarded with food upon successfully completing automated cognitive tasks (Gaffan et al., 1989; Fagot and Paleressompoulle, 2009; Gullstrand et al., 2022).

### 3.3. Electrophysiological measurements from retina, superior colliculus and visual cortex

#### 3.3.1. Retinal recordings

The retinal function can be assessed with an electroretinogram (ERG) which measures total retinal output based on photoreceptor functionality but is limited by its inability to detect signals from small areas of the retina, especially in transplantation experiments (Lin et al., 2017; Seiler et al., 2017). Multifocal ERG can record such local retinal responses but is not routinely used for rodent experiments. Using *ex vivo* retinal microelectrode arrays (MEAs), iPSC- and hESC-retina transplant-specific retinal ganglion responses to light stimulation were recorded in *rd1* mice and *Rho-S334ter-3* rats (Mandai et al., 2017; Watari et al., 2023). In addition, studies demonstrated that MEAs could detect light-evoked responses from transplanted human cone photoreceptors, confirming their functional integration into host retinal circuits. Transplanted cones derived from iPSC retinal organoids exhibited significant polarization and developed inner and outer segments essential for light detection (Ribeiro et al., 2021; Gasparini et al., 2022). These findings emphasize the utility of MEA technology in capturing localized retinal responses, offering a valuable tool for evaluating functional recovery and synaptic integration in retinal cell transplantation experiments.

#### 3.3.2. Superior colliculus (SC) recordings

However, retinal recordings are incapable of measuring the transplant’s ability to send visual signals to the brain, information which can be easily harvested from recording of visually evoked responses from SC topographic maps (Seiler and Aramant, 2012; Seiler et al., 2017). SC mapping is a technique commonly employed to study retinal degenerative diseases because, in rodents, most retinal ganglion cells (RGC) axons terminate in the SC. The SC is a part of the sub-cortical visual areas that contribute to optokinetic head tracking responses, can easily be accessed surgically, and has a well-known topographic retino-collicular map (Cang et al., 2018). RCS rats showed a topographic gradient decline of activity of SC which signified disease progression from the temporal to the nasal visual fields.

Transplantation of hESC-RPE into these rats demonstrated visually-evoked responses in SC areas corresponding topographically to the quadrant of retina transplantation (Thomas et al., 2016). Studies in hESC-derived retinal organoid transplants (McLelland et al., 2018; Lin et al., 2020) and co-grafts of hESC-derived retinal organoids with RPE (Thomas et al., 2021) have demonstrated, through SC recordings, similar topographical electrophysiological responses to light stimuli. Using a new multi-electrode array (MEA) system, now it is possible to conduct SC mapping more easily (Rajendran Nair et al., 2024), However, this approach has not yet been utilized in transplantation experiments.

#### 3.3.3. Recordings from the visual cortex

Single-cell recordings from the visual cortex have so far only been performed after fetal retinal transplants (Foik et al., 2018), not after hESC-retina transplants. The study showed that transplants rescue cortical visual responses and V1 l synaptic connectivity. For larger animal models, less invasive procedures are employed for vision assessments based on electrophysiology. Electrophysiological validation of the visual function in large animal models is conducted using ERG (Ofri, 2002; Gjorloff et al., 2004; Ekesten et al., 2013; Pasmanter and Petersen-Jones, 2020) and VEP (visual evoked potential) recording (Chang et al., 2022; Itoh et al., 2010; Maehara et al., 2018, 2020; Matsuo et al., 2018). Unlike SC recording, which is in general a terminal procedure conducted in rodents, both ERG and VEP used for larger animal models are not terminal and hence can be repeated in the same animal at different time points. The use of ERG is more appealing for preclinical studies since the technique is well-established and widely employed for human testing. Unlike ERG, VEP requires placement of the electrode over the skull bone and involves invasive procedures (Marenna et al., 2019; Liu et al., 2022a).

## 4. Assessment of morphological integration of the transplants using animal models

Synaptic connections between the transplant and host retina play a crucial role in the restoration of visual function and can be studied through various advanced techniques. Automated quantification approaches, such as QUANTOS (Akiba et al., 2019), have emerged as valuable tools for evaluating synaptic integration, providing precise and high-throughput analyses of synapse formation. Additionally, pre- and post-synaptic labeling was employed in studies like (Gasparini et al., 2022) to assess synaptic connectivity in detail. These advancements enable a more comprehensive understanding of transplant-host interactions and guide the course of visual restoration. Indeed, trans-synaptic tracing from the visually responsive sites in the SC to the fetal retinal transplant in the retina was studied using retrograde multisynaptic tracing with attenuated pseudorabies virus (PRV) (Seiler et al., 2008b), indicating synaptic connectivity between transplant and host interneurons. Labeling of different retinal layers of the retina was observed based on the post-injection time points. PRV labeling mainly occurred in the transplant retinal interneurons but rarely in the transplant photoreceptors.

With hESC-derived retinal transplants, synaptic connectivity was suggested by co-localization of donor label (SC121), host label (ratspecific antibody), and synaptophysin, using Imaris analysis (McLelland et al., 2018; Lin et al., 2020). Using genetically labeled donor photoreceptor terminals, Mandai et al. demonstrated direct contact between donor (mESC-retinal organoid) photoreceptor terminals and host rod bipolar cells (Mandai et al., 2017). Potential contacts between hESC-retina transplant photoreceptors and host bipolar cells were also found after transplantation in rd1 mice and *Rho-S334ter-3* rats by using Ribeye and Cx36 staining (Iraha et al., 2018; Tu et al., 2019). A genetic modification that reduced ON-bipolar cells in hESC-derived retinal organoids (knockout of Islet 1 gene) enhanced functional integration and synaptic connectivity after transplantation to *Rho-S334ter-3* nude rats (Yamasaki et al., 2022). These findings underscore the importance of both advanced imaging techniques and genetic approaches in improving our understanding of synaptic connectivity in retinal transplantation.

## 5. Conclusions and outstanding questions for future research

The retina and diseases affecting it, such as age-related macular degeneration (AMD) and retinitis pigmentosa (RP) are very complex. While significant advances have been made in the past decade, stem cell-based approaches have emerged as one of the most promising solutions for restoring vision However, critical hurdles, including immunological considerations, cell survival, and clinical translation, must be addressed to move in the field forward.

Even though the eye is considered immune-privileged due to its unique environment, there are still important immunological considerations, such as the risk of immune rejection or inflammation, particularly with allogeneic cell sources. Strategies like local immunosuppression or using autologous cells may help mitigate these risks. Enhancing the survival and functional integration of transplanted cells remains a priority. Techniques like advanced pre-differentiation protocols, scaffold-based systems, or genetic modifications can improve outcomes. Additionally, technologies such as QUANTOS for synapse quantification and imaging tools like Imaris are valuable for analyzing cell connectivity and integration (Akiba et al., 2024). While rodent models such as rd1 and RCS rats have been indispensable for preclinical research, their inability to replicate the human retina’s macula limits their translational relevance. Larger animal models, like pigs or non-human primates, provide better anatomical parallels but come with higher costs, ethical concerns, and logistical challenges. To bridge the gap between preclinical findings and clinical application, developing macula-like animal models and refining tools like trans-synaptic tracing are essential.

Moreover, the long-term safety and efficacy of stem cell therapies in humans need to be rigorously evaluated. Risks such as tumorigenicity, abnormal differentiation, and potential for unwanted proliferation or migration must be carefully monitored. Momentum in the field is growing, exemplified by BlueRock Therapeutics’ upcoming OpCT-001 trial for photoreceptor (PR) transplantation in 2025 (Halpern, 2025). This trial is expected to yield crucial data on the feasibility, safety, and functional restoration potential of PR transplantation in humans. If successful, OpCT-001 could establish a precedent for future cell-based therapies and unlock new avenues for treating retinal degeneration.

Emerging areas, such as extracellular vesicles (EVs) and the transfer of cytoplasmic material, represent pioneering directions that could significantly enhance graft survival and integration. EVs may facilitate intercellular communication and provide trophic support, while cytoplasmic material transfer could optimize host-graft interactions. Further exploration of these mechanisms could lead to breakthroughs in functional restoration and cell survival.

Several advancements are key to making PR transplantation a viable therapy. Standardized protocols must be established to generate functional PR cells with enhanced survival and integration. Advanced animal models with macular-like regions are critical to bridge the gap between preclinical studies and human trials. Innovative tools such as MEA recordings, QUANTOS-based quantification, and trans-synaptic tracing can refine transplantation techniques and improve our understanding of functional connectivity. Additionally, fostering collaboration among academia, industry, and clinical researchers is essential for translating laboratory findings into clinical trials.

Looking forward, the field’s most pressing challenges include scaling up stem cell production under GMP conditions, overcoming immune rejection, and refining minimally invasive delivery techniques. The delivery method of these cells, whether through subretinal, intravitreal, or other routes, also presents challenges in terms of achieving targeted delivery, appropriate cell integration, and minimal surgical complications. Subretinal delivery offers precise placement of cells in close proximity to degenerated areas but involves complex surgical procedures and risks such as retinal detachment or fibrosis. Intravitreal delivery, while less invasive, often leads to low integration efficiency and poor cell survival due to the longer distance cells must travel to reach target tissues. Innovative solutions, such as scaffold-supported delivery systems, offer mechanical stability and enhance the orientation of transplanted cells, facilitating better integration. For instance, biodegradable scaffolds like polycaprolactone and parylene-based membranes have shown promise in preclinical models, improving cell survival and structural support. Additionally, microfluidic delivery devices and advanced surgical instruments are being developed to enhance precision and reduce surgical complications.

To advance retinal regenerative therapies from experimental treatments to clinical reality, interdisciplinary collaboration among academia, industry, and clinical researchers is essential. Standardized protocols, advanced imaging technologies for real-time monitoring of graft placement and integration, and translational research must align to ensure accessibility, scalability, and long-term efficacy. Ultimately, these efforts have the potential to transform retinal therapy, offering hope and improved quality of life to millions of patients worldwide.

## Figures and Tables

**Fig. 6.**
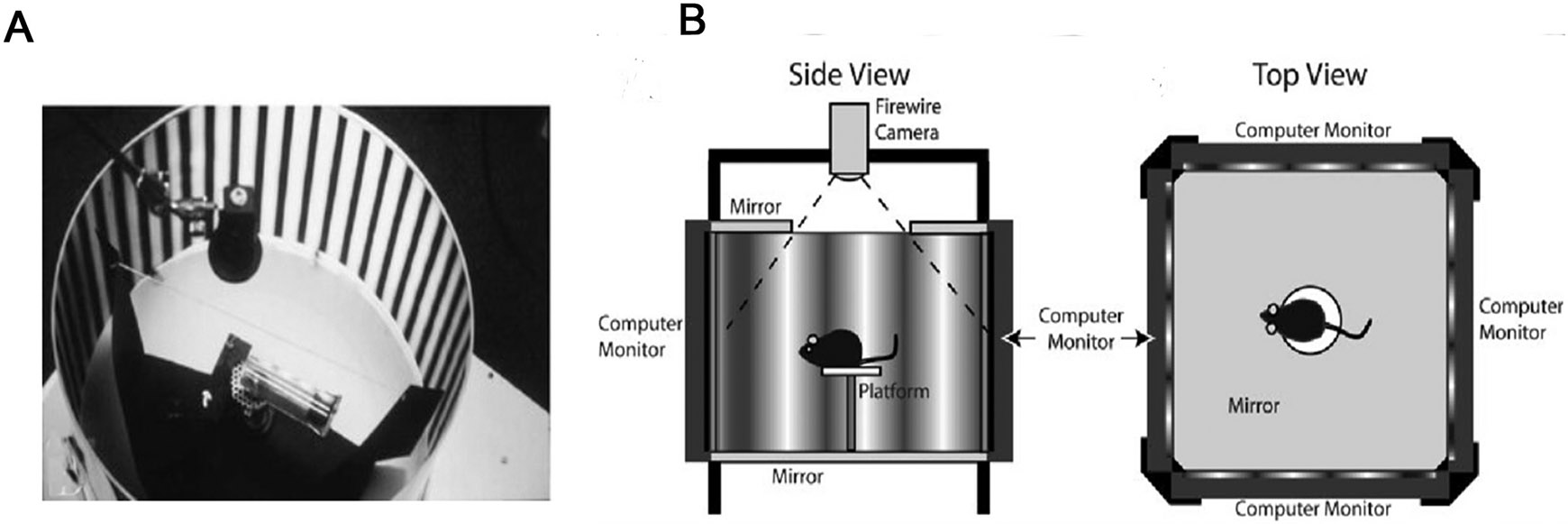
OKN Testing Instruments for Vision Assessments in Rodents. A. Traditional set-up utilizing a rotating drum. The image depicts a top-down view of the drum, including the video camera used to record head movements, the stationary black wall surrounding the drum, and the rat holder positioned at the center. (Reproduced from (Thomas et al., 2004b), Thomas et al. Optokinetic test to evaluate visual acuity of each eye independently, Journal of Neuroscience Methods 138 (1–2):7–13)). B. Schematic representation of the optomotor testing apparatus. (A) Side view. A mouse is placed on a platform positioned in the middle of an arena created by a quad-square of computer monitors. Sine wave gratings drawn on the screens are extended vertically with floor and ceiling mirrors. A video camera is used to monitor the animal’s behavior from above. (B) Top view. The mouse is surrounded by 360° of gratings and is allowed to move freely on the platform. (Reproduced from (Prusky et al., 2004): Prusky et al. Rapid quantification of adult and developing mouse spatial vision using a virtual optomotor system, *Invest Ophthalmol Vis Sci* 45 (12):4611–6).

**Table 1:** Small animal models for retinal diseases.

Animal Model	Disease Model	Modification	Affected Cell	References
Complement Factor H (*Cfh*)Knockout mice	AMD	Absence of CFH	RPE PRs	(Hoh Kam et al., 2016; Sridevi Gurubaran et al., 2021; Crowley et al., 2023)
*CFH* −/− *Y402H* mice	AMD	Single nucleotide polymorphism at amino acid position 402	RPE	Landowski et al. (2019)
*Ccl2* −/− or *Ccr 2* −/−mice	AMD	Knockout of CC-motif ligand or receptor	PRs	Badia et al. (2021)
*CIB2* deficient mice	AMD	Under-expression of calcium and integrin binding protein 2	RPE	Sethna et al. (2021)
CD36 −/− mice	AMD	CD36 knockout (principal receptor of oxidized phospholipid)	RPE; micro-vascular endothelial cells; macrophage	Mellal et al. (2019)
rd1 mice (C3H/HeJ, MHC-mismatched)	Retinitis Pigmentosa (RP)	Mutation in the Pde6b gene	Rod photoreceptors	Uyama et al. (2022)
rd1 mice (PDE6β^rd1/rd1)	Retinitis Pigmentosa (RP)	PDE6β mutation leading to photoreceptor loss	Rod photoreceptors	(Barnea-Cramer et al., 2016, 2020)
rd1 mice (PDE6β^rd1/rd1)	Retinitis Pigmentosa (RP)	PDE6β mutation leading to photoreceptor loss	Rod photoreceptors	(MacLaren et al., 2006; Barber et al., 2012;Pearson et al., 2016)
rd1/Foxn1^nu mice	Retinitis Pigmentosa (RP)	PDE6β mutation (rd1) + Foxn1^nu (immunodeficient)	Rod photoreceptors, host	Ribeiro et al. (2021)
*Rd12* mice	Retinitis pigmentosa	Mutation in Rpe65 gene	retina is immunodeficient PRs	(Wright et al., 2014; Brown et al., 2022)
*Rd10* mice	Leber’s congenital amaurosis (LCA)	r-PDE mutation that leads to rod degeneration	Rods; microglia activation	Wang et al. (2018)
*Rho*−/− mice	Retinitis pigmentosa	Rhodopsin knockout	Rod function	Tucker et al. (2011)
Rhodopsin Pro23His (P23H) transgenic mice	Retinitis Pigmentosa	Proline to Histidine substitution at codon 23 in rhodopsin gene	PRs	Liu et al. (2022c)
*BKS-Lepre*^*m2Cd479/Gpt*^ mice	Diabetic retinopathy (DR)	Homozygous spontaneous mutation in Leprdb which demonstrates diabetic symptoms	PRs	Ai et al. (2022)
Oxygen Induced retinopathy (OIR) mice	DR	Exposure of hyperoxic conditions to pups during retinal vasculature development	PRs	(Xu et al., 2020; Yazdanyar et al., 2020;Cheung et al., 2021)
*ELOVL4* ± mice	Stargardt type 3	Mutation in the ELOVL4 gene disturbs development of PR Outer Segment membranes	PRs RPE	(Kuny et al., 2015; Mazzilli et al., 2020)
Cpfl1 mouse	Cone Degeneration	Mutation in Pde6c gene	Cone photoreceptors	Gasparini et al. (2022)
NMDA mice	Retinal ganglion cell depletion	NMDA-induced retinal ganglion cell	Retinal ganglion cells	Chen et al. (2022)
Glaucoma mouse models	Glaucoma	Intraocular pressure elevation causing damage to retinal ganglion cells	Retinal ganglion cells	Mead et al. (2018)
*RCS* rats	Retinitis Pigmentosa	Deletion in MERTK gene	RPE cells	(Thomas et al., 2016; Zou et al., 2019;Shrestha et al., 2020a; Salas et al., 2021; Liang et al., 2022)
*RCS nude (Hsd:RH-Foxn1rnu)* rats	Retinitis Pigmentosa	Deletion in *MERTK* gene null mutation in *Foxn1*	RPE cells T cells	(Lin et al., 2020; Rajendran Nair et al.,2021;Thomas et al., 2021)
*Rho-S334ter* rats	Retinitis Pigmentosa	Stop codon at position 334 in *Opsin* protein	PR cells	(Laver et al., 2015; LaVail et al., 2018)
*SD-Foxn1 Tg(S334ter) 3LavRrrc* nude rats (RD nude rats)	Retinitis Pigmentosa	Stop codon at position 334 in *Opsin*protein null mutation in *Foxn1*	PR cells T cells	(McLelland et al., 2018; Tu et al., 2019;Watari et al., 2023)
*Rho-P23H* rats	Retinitis Pigmentosa	Proline to Histidine substitution at codon 23 in *Opsin* protein	PR cells	Laver et al. (2015)
NMDA rat model of vision loss	Retinal ganglion cell depletion	Intravitreal injection of NMDA causing excitotoxicity	Retinal ganglion cells	Eastlake et al. (2019)
Retinal ischemia rat models	Retinal ischemia	Ischemia induced in retinal tissue	Photoreceptor cells, Retinal ganglion cells	Mathew et al. (2019)
Optic nerve crush rat models	Optic neuropathy	Crushing of the optic nerve to simulate optic nerve injury	Retinal ganglion cells	Cui et al. (2021)
Sprague-Dawley rats	Geographic Atrophy-like Pathology	Subretinal injection of NaIO_3_	RPE and Photoreceptors	Naik et al. (2024)
C57BL/6 J mouse	Retinal Degeneration	systemic injection of NaIO_3_	RPE and Photoreceptors	Anderson et al. (2025)
Brown Norway rat	Age-Related Macular Degeneration (AMD) Model	Sublingual vein injection of NaIO_3_	RPE and Photoreceptors	Koster et al. (2022)
C57BL/6 J mouse	Localized Retinal Degeneration	Laser-induced retinal injury	RPE and Photoreceptors	(Lorach et al., 2015; Ibbett et al., 2019)
13-lined ground squirrel	Retinal damage	Injection of ATP or retinal detachment	PR cells	Yu et al. (2024)

**Table 2:** Large animal models for retinal diseases.

Animal Model	Disease Model	Modification	Affected Cell	References
Induced-retinal degeneration albino rabbits	Geographic Atrophy (late-stage dry AMD)	Injection of NaIO_3_ into the subretinal space	RPE cells	(Petrus-Reurer et al., 2017, 2018; Tay et al., 2023)
TgP347L transgenic rabbit	RP	Rhodopsin Pro347Leu transgene expression	PRs	Jones et al. (2011)
Large-eyed rabbit model	GA	Injection of NaIO_3_ into the subretinal space	RPE, PRs	Petrus-Reurer et al. (2017)
Rabbit	Retinal detachment	Injection of 0.1 ml balanced salt solution under the retina to induce retinal detachment	PR; Bipolar cells Müller cells Amacrine and horizontal cells	Barliya et al. (2017)
Cat	RP	Intravitreal ATP injection	PRs	Aplin et al. (2016)
Abyssinian (and Somali) Cat	Autosomal Recessive (AR) progressive rod-cone degeneration (rdAc)	CEP290 gene mutation (IVS50 + 9 T > G)	PRs (Rods and Cones)	(Menotti-Raymond et al., 2007; Minella et al., 2018, 2023; Narfstrom et al., 2009; Seiler et al., 2009; Winkler et al., 2020)
Cat	LCA	Genetic mutation (AIPL1)	PRs	(Rah et al., 2005; Moshiri, 2021)
Cat	Fundus Albipunctatus	Genetic mutation (RDH5)	PRs	(Moshiri, 2021; Occelli et al., 2022)
Cat	RP	Genetic mutation (CRX^rdy^)	PRs (no recordable cone ERG)	(Occelli et al., 2016; Singh et al., 2019)
Dog	RP	Genetic Mutation (PDE6A)	PRs	(Petersen-Jones et al., 1999; Pasmanter et al., 2021)
Dog	RP	Genetic Mutation (PDE6β)	PRs	Pichard et al. (2016)
Dog	RP	Genetic Mutation (CNGB1)	PRs	Pasmanter et al. (2021)
Dog	RP	Genetic Mutations (crd 2/NPHP5, xlpra2/RPGR)	Retinal Vasculature	Ripolles-Garcia et al. (2022c)
Dog	RP	Genetic mutation (CNGB1)	PRs	Winkler et al. (2013)
Dog	RP	rcd1/PDE6B mutation	PRs	Ripolles-Garcia et al. (2022b)
Dog	Acute Rod Degeneration	Acute Light-Induced Degeneration (RHOT4R/+)	PRs, retinal Vasculature	Ripolles-Garcia et al. (2022a)
Dog	Stargardt Maculopathy	Genetic Mutation (ABCA4)	PRs	Makelainen et al. (2019)
Dog	Leber Congenital Amaurosis (LCA)	Genetic Mutation (RPE65)	RPE PRs	Acland et al. (2001)
Dog	XLPRA	Genetic mutation (RPGR)	PRs	Zhang et al. (2002)
Dog	Best Vitelliform Macular Dystrophy	Genetic mutation (BEST1)	RPE	(Guziewicz et al., 2018; Winkler et al., 2020)
Dog (German Spitz)	Progressive Retinal Atrophy (PRA)	Genetic Mutation (GUCY2D, frameshift mutation)	PRs	Bortolini et al. (2023)
Dog (Dachshound)	Neuronal ceroid lipofuscinosis	Null mutation of tripeptidyl peptidase-1 (TPP1), lysosomal storage disease	Thinning of all retinal layers, resulting in blindness	Tracy et al. (2016)
Pig	RPE damage	Mechanical damage (RPE debridement)	RPE PRs	Del Priore et al. (1995)
Pig	RP	Rhodopsin Pro347Leu transgene expression	PRs (rods and cones); Müller cells	(Petters et al., 1997; Blackmon et al., 2000; Ghosh et al., 2004; Sommer et al., 2011b; Klassen et al., 2012)
Pig	autosomal dominant RP	Pro23His (P23H) Rhodopsin transgene	photoreceptors	(Scott et al., 2014, 2017)
Pig	cone dystrophy	dominant mutant allele of the guanylate cyclase 2D (GUCY2D) gene	Cone PRs	Kostic et al. (2013)
Pig	Usher’s syndrome	human mutation USH1C gene	PR ciliopathy	Grotz et al. (2022)
Pig	Stargardt-like macular degeneration	ELOVL4 Transgene expression	Photoreceptors (slow RD)	Sommer et al. (2011a)
Pig	AMD	Laser-induced RPE injury	RPE	(Sharma et al., 2019; Barone et al., 2023)
Minipig	Geographic atrophy	Injection of sodium iodate (NaIO_3_)	RPE cells	Duarri et al. (2021)
NHP	Bardet-Biedl Syndrome (BBS) Retinal Atrophy	Genetic mutation (BBS7)	Retinal vasculature	(Peterson et al., 2019; Winkler et al., 2020)
NHP	Achromatopsia (ACHM)	Genetic mutation (PDE6C)	Cone PR Cells	(Moshiri et al., 2019; Winkler et al., 2020)
NHP	Retinal Ischemia/Reperfusion Injury (RI/RI)	Induced High Intraocular Pressure	Retinal Ganglion Cells	Gong et al. (2023)
Macaque (nonhuman primate)	Retinal degeneration	Selective ablation of host photoreceptors using an ultrafast laser	Host photoreceptors	Aboualizadeh et al. (2020):
Cynomolgus Monkey	Focal selective photoreceptor degeneration	Induced by subretinal injection of cobalt chloride	PRs	Shirai et al. (2016)
Cynomolgus Monkey; Rhesus Monkey	Focal selective photoreceptor degeneration	Induced by 577-nm optically pumped semiconductor laser photocoagulation	PRs	(Shirai et al., 2016; Tu et al., 2019; Uyama et al., 2022)
Monkey (Cynomolgus Macaque)	Local RPE degeneration	Laser ablation of RPE (450–550 mW laser)	RPE	Kajita et al. (2024)

**Table 3:** List of immunosuppressants used for cell replacement therapies in animal models.

Name of the drug	Animal model used	Mode of administration	dosage	reference
Ozurdex (Dexamethasone)	Rhesus monkeys (*Macaca mulatta*)	IVT	0.7 mg (usual dose)	Xian et al. (2019)
Ozurdex	Pig	Intravitreal capsule	0.7 mg (usual dose)	Koss et al. (2016)
Rapamycin (RAP)	Rhesus monkeys	IVT	90 μg/eye/week	Xian et al. (2019)
Cyclosporine A	Monkey	Oral mixed with food	10 mg/kg/day	Fujii et al. (2020)
Triamcinolone	Monkey	Sub-Tenon injection	4 mg total dose	Fujii et al. (2020)
Triamcinolone	Rat	IVT	2–4 mg per eye (Regular Dose)	Singhal et al. (2010)
Triamcinolone Acetonide	Cpfl1 mouse model	Monthly IVT	80 μg/μL per injection, monthly	Gasparini et al. (2022)
Triamcinolone (in combination with Cyclosporine-A	Rabbit	IVT injection	2 mg	Tay et al. (2023)
Cyclosporine A (in comb. w. Triamcinolone)	Rabbit	oral	20 mg/kg/d	Tay et al. (2023)
Dexamethasone (in comb. w. Cyclosporine A)	Rat (RCS)	IP Injection	1.6 mg/kg daily for 2 weeks	Cooper et al. (2016)
Cyclosporine A (in comb. w. Dexamethasone)	Rat (RCS)	Oral (drinking water)	210 mg/ml	Cooper et al. (2016)
Tacrolimus (FK-506; TAC)	BALB/c mice	Oral Gavage	4 mg/kg/day	Pearl et al. (2011)
Sirolimus (SIR)	BALB/c mice	Oral Gavage	3 mg/kg/day	Pearl et al. (2011)
Anti-CD40L (MR-1)	BALB/c mice	I.P.	20 mg/kg on days 0, 2, 4, and 6	Pearl et al. (2011)
Anti-LFA-1 (M17/4)	BALB/c mice	I.P.	20 mg/kg on days 0, 2, 4, and 6	Pearl et al. (2011)
CTLA4-Ig	BALB/c mice	I.P.	20 mg/kg on days 0, 2, 4, and 6	Pearl et al. (2011)
PD-L1 (Programmed Death-Ligand 1)	Hu-mice	Genetically modified hESCs expressing PD-L1 were transplanted subcutaneously into humanized mice (Hu-mice).	Not applicable	Rong et al. (2014)
Prednisolone	RCS Rat	Oral	5 mg/l in drinking water	Singhal et al. (2008)
Cyclosporine A	RCA Rat; Neonatal Lister hooded rats	Oral administration (in drinking water)	210 mg/l of drinking water	Singhal et al. (2008)
Azathioprine	RCS rats; Neonatal Lister hooded rats	Oral administration (in drinking water)	20 mg/l of drinking water	Singhal et al. (2008)
Tacrolimus (FK-506; TAC) in combination with mycophenolate mofetil (MMF)	Immunocompetent *Rho-S334ter* rats	Subcutaneous implantation of TAC pellet;MMF in food	TAC: 20 mg 90 d release pellet; MMF: 300 mg/kg MMF in food	Sims et al. (2025)

**Table 4:** List of immunodeficient animal models used for testing cell replacement therapies.

Animal model	Disease condition	Cell replacement therapy	references
Rabbit (X-SCID)	Retinal Injury	human adipose-derived mesenchymal stem cells (AD-MSCs) and bone marrow MSCs (BM-MSCs)	Kim et al. (2021)
Nude Mice, SCID mice, NOD-SCID mice, NOG mice	None (normal retina); immunodeficient	iPSC-derived RPE	Kanemura et al. (2014)
NOG-rd1-2 J Mouse	RP, immunodeficient	Transplantation of human ESC-derived retinal sheets	Iraha et al. (2018)
NOG-rd10 Mouse	RP, immunodeficient	Transplantation of human ESC-derived retinal sheets	Iraha et al. (2018)
NOD/SCID/IL-2Rgc^null^ (NSG) mice	None (normal retina); immunodeficient	GMP-grade human iPSC-derived retinal pigment epithelial cells	Zhang et al. (2021)
IL2rγ^−/−^ mice; Crx^tvrm65^/IL2rγ^−/−^ mice	Deletion of IL2rγ; Crx-mutation: lack of phototransduction	hESC-derived photoreceptors	Zhu et al. (2017)
Immunodeficient rnu *(foxn1*−/−) nude rat	None (normal retina);immunodeficient	hESC-RPE cells	Diniz et al. (2013)
		Human embryonic stem cell (hESC)-derived retinal tissues	(Shirai et al., 2016; Watari et al., 2023)
RD Nude Rat (*SD-Foxn1 Tg* *(S334ter)3Lav*)	PR degeneration (RP);immunodeficient	hESC-derived neural progenitor cells	Seiler et al. (2014)
		Retinal Sheets (from transgenic rats expressing human placental alkaline phosphatase)	Seiler et al. (2017)
		Sheets of human fetal retina and RPE	Lin et al. (2018)
		hESC-derived retinal organoids (ROs)	(Shirai et al., 2016; McLelland et al., 2018; Tu et al., 2019; Uyama et al., 2022)
RD Nude Rat (*SD-Foxn1 Tg (S334ter)3Lav*)	PR degeneration (RP); immunodeficient	Monkey (Cynomolgus macaque)-iPSC-derived ROs	Uyama et al. (2022)
Immunodeficient RCS rat	RPE dysfunction (*MerTK* −/−); immunodeficient	hESC-RPE, iPS-RPE cell suspension	Thomas et al. (2018)
		hESC-derived retinal organoid sheets	Lin et al. (2020)
		hESC-derived retinal organoid sheet combined with RO sheet (“Co-graft”)	Thomas et al. (2021)
NSG mice, RCS rat	Normal retina (NSG mice); RPE dysfunction (*MerTK* −/−) (RCS rat)	Human iPSC-derived RPE cells	Zhang et al. (2021)
MHC Homozygote monkey (Cynomolgus macaque)	none (normal retina)	Monkey iPSC-derived RPE cells (MHC matched and mis-matched)	Sugita et al. (2016)

**Table 5:** List of animal models used for demonstrating surgical feasibility.

Animal model	Cell type transplanted	Mode of administration	Technique used	References
Chemically induced photoreceptor degeneration mouse model	Dissociated retinal cells from neonatal Nrl-GFP transgenic mice	Subretinal injection	Microsurgical	Suzuki et al. (2007)
Immunosuppressed *RCS* rat	Human ESC-derived RPE	Subretinal injection	A syringe with a blunt needle	da Cruz et al. (2007)
*RCS* rat, *Elov14* mouse, *NIH III* immune-deficient mouse	hESC-derived RPE cells	Subretinal transplantation	Micropipette	Lu et al. (2009)
Immunosuppressed *RCS* rat	hESC-RPE cells and parylene substrate coated with vitronectin	Subretinal transplantation	33-gauge steel needle	Thomas et al. (2016)
immunosuppressed *RCS* rat, Rd1 mouse,	Eye-wall c-kit+/SSEA1− cells	Subretinal	Microsurgical injection	Chen et al. (2016a)
Immunosuppressed *RCS* rat	hESC-derived RPE cells	Subretinal implantation	Not specified in the text, but typically a microsyringe or custom implantation tool would be used	Ribeiro et al. (2013)
NOG mice, Nude Rats	iPSC-derived RPE cells, iPSC cells, and HeLa cells	Subretinal	collagen-lined sheet (subretinal transplantation); Matrigel (for embedding cells during subcutaneous injections)	Kanemura et al. (2014)
Nude rats	hiPSC-derived RPE strips	Subretinal	Micropipette	Nishida et al. (2021)
Nude rats	hiPSC-derived RPE	Subretinal	Microsyringe, sheet-based transplantation	Li et al. (2022)
*rd1* Mouse (PDE6βrd1/rd1)	rod photoreceptor precursor cells	Subretinal injection	Micropipette	(MacLaren et al., 2006; Barber et al., 2013; Pearson et al., 2016)
*rd1/Foxn1nu Mouse*	Human pluripotent stem cell-derived cone photoreceptors	Subretinal injection	Fluorescence-activated cell sorting (FACS) and micropipette	Ribeiro et al. (2021)
*rd1* Mouse	photoreceptor progenitors derived from human ESCs and iPSCs	Subretinal	Microinjection	(Barnea-Cramer et al., 2016, 2020)
rd1 Mice (C3H/HeJ, MHC-mismatched)	PSC-derived retinas	Subretinal injection	Micropipette	Uyama et al. (2022)
*rd10* mouse	hESC-derived photoreceptor progenitors	Subretinal	Microinjection	Tun et al. (2023)
Cpfl1 Mouse	PSC-derived cone photoreceptor precursors.	Subretinal injection	FACS sorting and micropipette	Gasparini et al. (2022)
RCS rat	Human RPE cells	Subretinal	Microinjection	Pinilla et al. (2007)
RCS rat	Human iPSC-derived RPE and PRP	Trans-scleral subretinal injection	Microinjection	Surendran et al. (2021)
RCS rat	Human iPSC-derived RPE and RPC	Trans-scleral subretinal injection	Microinjection	Salas et al. (2021)
RCS rat	hESC-derived RPE cells	Subretinal implantation	Ultrathin Substrate Implantation Tool	Hu et al. (2012)
RCS Rat	iPS-derived RPE cells	Subretinal injection	Hamilton syringe with a 30-gauge needle	Carr et al. (2009)
Immunodeficient RCS rat	hESC-RPE, iPS-RPE	Subretinal	Injection	Thomas et al. (2018)
Immunodeficient Nude Rat	hESC-RPE cells	Subretinal	Microsyringe pump	Diniz et al. (2013)
Rodents (Rat, Mouse)	RPE	Subretinal Injection	Syringe with blunt needle	(Westenskow et al., 2015, 2016)
Rat retinal degeneration model (induced by sodium iodate injection)	hESC-RPE	Subretinal injection	Microsyringe	Park et al. (2011)
*Rho-S334ter*-line-5 transgenic rats	Rat fetal retinal sheets	Subretinal injection	custom implantation instrument	Thomas et al. (2004a)
*SD-Foxn1 Tg(S334ter)3Lav* (RD nude) Rat	hESC-derived neural progenitor cells	Trans-scleral subretinal injection	custom implantation instrument	Seiler et al. (2014)
*Rho-S334ter-3* rat	Rat fetal retinal sheets (expressing human placental alkaline phosphatase (hPAP)	Subretinal transplantation	custom implantation instrument	(Seiler et al., 2010; Foiket al., 2018)
Immunodeficient *Rho-S334ter*Line-3 Rat (RD nude rat)		Subretinal transplantation	custom implantation instrument	Seiler et al. (2017)
	Human fetal retinal sheets	Subretinal transplantation	custom implantation instrument	Lin et al. (2018)
	Retinal organoid sheets	Subretinal transplantation	custom implantation instrument	(McLelland et al., 2018; Lin et al., 2024)
RCS rat	Retinal organoid sheets	Subretinal transplantation	custom implantation instrument	Lin et al. (2020)
Rabbit	Cultured RPE monolayer	Subretinal transplantation	A syringe with a blunt needle	Al-Nawaiseh et al. (2016)
Rabbit	hiPSC-derived RPE strips	Subretinal injection	24-gauge indwelling needle cannula	Nishida et al. (2021)
Swine (Minipig)	Human iPSC-derived RPE	Subretinal	Custom syringe with blunt needle	Duarri et al. (2021)
Rhodopsin Pro347Leu transgenic pig	Full-thickness neuroretinal graft (pig)	Subretinal injection/transplantation (after vitrectomy and retinotomy)	Microsyringe or surgical tools for transplantation	Ghosh et al. (2004)
Rhodopsin Pro347Leu-transgenic pig	RPC	Subretinal injection	Microsyringe or surgical tools for transplantation	Klassen et al. (2012)
Miniature pig	iPSC-RPE cells	Subretinal injection	Microsyringe or surgical tools for transplantation	Sohn et al. (2015)
Abyssinian cat	fetal retinal sheet (cat allograft)	Subretinal injection (transplantation)	Thin-walled glass cannula connected to Hamilton syringe	Occelli et al. (2021)
*CrxRdy/*+ cat	Retinal organoids	Subretinal injection	Thin-walled glass cannula connected to Hamilton syringe	Occelli et al. (2021)
Dog	Photoreceptor precursor cells from CRX-TdTomato expressing retinal organoids	Subretinal injection	Subretinal injector (RetinaJect, SurModics, CA)	Ripolles-Garcia et al. (2022b)
Squirrel monkey	hESC-derived neuronal cells	Subretinal injection	iTRACK-275 microcatheter	Chao et al. (2017)
MHC Homozygote monkeys	iPSC-derived RPE cells	Subretinal injection	Injection cannula	Sugita et al. (2016)

**Table 6:** Visual function evaluation tools or techniques after cell transplantation in small animals.

Animal Model	Evaluation Technique	Purpose/Application	Reference
Royal College of Surgeons (RCS) rats	Head tracking, Pattern discrimination, Single-unit cortical physiology	Evaluate the preservation of cortically mediated vision after RPE cell transplantation in a model of retinal degeneration	Coffey et al. (2002)
*Rho-S334ter-line-5* rat (slow RD)	SC electrophysiology	Assess if retinal transplants preserve SC visual responses	Thomas et al. (2004a)
RCS rats	ERG, Histology, IHC	Assess synaptic connectivity and photoreceptor function preservation after RPE cell transplantation	Pinilla et al. (2007)
Chemically induced photoreceptor degeneration mouse model (MNU)	Histology; Electroretinography (ERG)	Examine the survival, distribution, and integration of transplanted photoreceptor cells in the host retina	Suzuki et al. (2007)
*Rho-S334ter* line 3 rhodopsin retinal degenerate rats	Optokinetic head-tracking, SC electrophysiology, immunohistochemistry	Evaluate the functional efficacy of retinal progenitor cell (RPC) sheets with BDNF microspheres following subretinal transplantation	Seiler et al. (2008a)
*Rho-S334ter* line 3 rhodopsin retinal degenerate rats	SC electrophysiology, transsynaptic PRV tracing, immunohistochemistry, electron microscopy	Investigate synaptic connectivity of retinal transplants responding to light	Seiler et al. (2008b)
RCS rats, Elov14 mice, NIH III mice	Optomotor response, SC luminance threshold, Histology	Assess visual acuity, sensitivity, photoreceptor preservation, and safety (tumorigenic potential) after hESC-RPE transplantation	Lu et al. (2009)
RCS Rats	Optokinetic Testing (Head-Tracking Response), Light-Induced c-Fos Expression	Assess the preservation of visual function and neuronal activity in the retina after iPS-RPE cell transplantation	Carr et al. (2009)
*Rho-S334ter-3* line 3 rhodopsin retinal degenerate rats	Confocal microscopy, EM, Electrophysiology (SC)	Assess synaptic connections and visual restoration post-transplantation of rat retinal sheets expressing hPAP	Seiler et al. (2010)
Rat Retinal Degeneration Model (NaIO_3_-induced), RD Nude Rats	Histology, IHC, RT-PCR	Assess cell survival and integration post-hESC-RPE transplantation	Park et al. (2011)
RCS rats, Copenhagen rats	SD-OCT, Histology	Evaluate the feasibility, placement accuracy, and cell retention of implanted ultrathin substrates with hESC-RPE monolayers in the subretinal space	Hu et al. (2012)
RCS Rat	ERG, OKN, SC Recording, Histology, Tumorigenicity Testing	Assess retinal response, visual tracking, neural activity, retinal structure, and evaluate tumorigenicity after hESC-RPE transplantation	Diniz et al. (2013)
RCS rats	NIR imaging, Histology, Immunohistochemistry	Detect the presence and survival of hESC-RPE cells implanted on a parylene membrane	Ribeiro et al. (2013)
RCS rats	Optokinetic response (OKR), Electrophysiological recordings, retinal histology	Assess the impact of iNPC transplantation on disease progression, retinal preservation, and visual function in AMD model	Tsai et al. (2015)
RCS rats	Optical Coherence Tomography (OCT), Optokinetic Testing (OKT), Superior Colliculus (SC) Electrophysiology, Histology	Assess RPE implant placement, visual function, cell survival, and integration	Thomas et al. (2016)
Gnat 1−/− mice (rod functionde-ficient, congenital stationary night blindness model)	Optokinetic head tracking, visually guided behavior, intrinsic imaging, synaptic connectivity analysis	To demonstrate the ability of transplanted rod-precursor cells to restore functional rod-mediated vision and synaptic integration in the retina	Pearson et al. (2012)
*rd1* mice	ERG, Light/Dark transition test, Histology	Assess retinal function, synapse formation, and behavioral changes post-transplantation of eye wall progenitor cells	Chen et al. (2016b)
*rd1 mice*	Microelectrode Array (MEA)	Evaluate transplant-specific retinal ganglion responses and light-evoked responses after photoreceptor transplantation	(Mandai et al., 2017; Ribeiro et al., 2021; Akiba et al., 2024)
*Cpfl1 mice*	Microelectrode Array (MEA)	Assess functional integration of transplanted photoreceptors and light-evoked responses in cone-degenerate models	Gasparini et al. (2022)
*rd1* mice	OMR, Light avoidance test, Histology	Assess visual function recovery and photoreceptor integration after transplantation of human PhRPs derived from ESCs and iPSCs	Barnea-Cramer et al. (2016)
*NOG-rd1-2J, NOG-rd10* mice	Retinal MEA, whole-mount immunostaining	Evaluate the long-term survival, maturation, and functional integration of human ESC-derived retinal sheets in mouse models of end-stage retinal degeneration	Iraha et al. (2018)
*B6.CXB1-Pde6b^rd10^/J* mice (rd10 mice)	Full-field ERG, Water maze swimming test, Histology	Assess survival, maturation, and visual improvement following photoreceptor progenitor transplantation	Tay et al. (2023)
Mice (NaIO_3_-induced blindness)	Optokinetic head tracking, Light avoidance assays	Evaluate vision restoration after co-transplantation of RPE and photoreceptors in a hydrogel	Mitrousis et al. (2020)
*RhoP23H/*+ mice (retinal degeneration model)	Optokinetic response (OKR), histology	Evaluate the effects of subretinal transplantation of various cell types on retinal degeneration and visual function improvement	Liu et al. (2020)
Immunodeficient RCS Rats (RCS-*p*+*/Foxn1mu*)	OCT, ERG, OKN, SC, Histology	Assess retinal response, visual tracking, neural activity, and retinal structure after hESC-RPE transplantation	Thomas et al. (2018)
Immunodeficient *Rho-S334ter* Line-3 Rat (RD nude rat)	OCT, OKN, ERG, SC Electrophysiology	Test retinal sheet transplants for restoring visual function in retinal degeneration	Seiler et al. (2017)
Immunodeficient *Rho-S334ter*-3 rats (RD nude rats)	Optokinetic testing, Superior colliculus electrophysiology, Immunohistochemistry, OCT	Assess differentiation, integration, and visual function improvement of transplanted hESC-derived retina organoid sheets	(McLelland et al., 2018; Lin et al., 2024)
Immunodeficient *Rho-S334ter* Line-3 Rat (RD nude rat)	Retinal MEA, immunohistochemistry	Evaluate the impact of ISL1 gene knockout in human ESC-derived retinal organoids on enhancing synaptic connectivity and functional integration with the host retina after transplantation	Yamasaki et al. (2022)
Immunodeficient *Rho-S334ter* Line-3 Rat (RD nude rat)	Electrophysiology-retinal multielectrode array (MEA), Optical Coherence Tomography (OCT), Histology; whole-mount immunostaining	Assesses functional integration of transplanted ESC-and iPSC-derived retinal organoid sheets and RO-derived retinal progenitor cell reaggregates; record light-evoked retinal ganglion cell responses	(Mandai et al., 2017; Tu et al., 2019; Watari et al., 2023; Iwama et al., 2024)
Immunodeficient RCS *rats*	OCT, Optokinetic behavioral testing, ERG, Superior colliculus electrophysiology, IHC analysis	Assess survival, integration, and visual function improvement of transplanted human embryonic stem cell-derived retinal organoid sheets	Lin et al. (2020)
PDE6b KO rat (no immunosuppression)	Spectral domain OCT, ERG, Histology	Evaluate survival and functional effect after hiPSC-derived retinal cell transplantation	Yang et al. (2021)
Immunodeficient RCS rats	Histology, OCT, Optokinetic behavioral testing, Superior colliculus electrophysiology	Assess structural reconstruction, visual function improvement, and integration of co-grafted retinal organoid and RPE sheets	Thomas et al. (2021)
RCS rats, *NOD.SCID-rd1* mice	ERG, Synaptic Markers, Histology, IHC, Optokinetic Tracking, Light Perception	Assess photoreceptor function, connectivity, and visual improvement after RPE/PRP transplantation	Surendran et al. (2021)
RCS Rat	ERG, Histology, Visual Tests (Optokinetic Tracking)	Assess photoreceptor function, ONL preservation, and visual function improvement after combined RPE and RPC transplantation	Salas et al. (2021)

**Table 7:** Visual function evaluation tools or techniques after cell transplantation in large animals.

Animal Model	Evaluation Technique	Purpose/Application	Reference
MHC Homozygote monkeys (cynomolgus monkeys)	Optical Coherence Tomography (OCT), Fundus Photography, Fluorescein Angiography (FA)	Assess the survival and integration of iPS-RPE cells, monitor immune rejection, and evaluate retinal structure post-transplantation	Sugita et al. (2016)
Rabbit	Spectral domain optical coherence tomography (SD-OCT), histology	Assess integration and success	Al-Nawaiseh et al. (2016)
Rabbit	Histology	Evaluate synaptic connectivity of engrafted cells with degenerated host retina	Tay et al. (2023)
Minipigs (Swine) with NaIO_3_-induced RPE damage	SD-OCT, FAF, Histology, Immunohistochemistry	Assess hiPSC-RPE transplantation in a GA model	Duarri et al. (2021)
*Rhodopsin Pro347Leu* transgenic pigs	Immunohistochemistry, Histology, Fluorescence microscopy	Evaluate survival and differentiation of retinal progenitor cells (RPCs) after transplantation	Klassen et al. (2012)
Abyssinian cats	Fundus examinations, ERGs, Angiography, Histology, Immunohistochemistry	Assess integration and lamination of fetal retinal sheet transplants in degenerating retina	Seiler et al. (2009)
Miniature Pig	Immunohistochemistry, Histology, Vitreous cytokine analysis	Assess immune response to iPSC-derived RPE cell transplants in subretinal space	Sohn et al. (2015)
Cynomolgus monkey, Rhesus monkey	Visual guided saccade (VGS), OCT imaging, immunohistochemistry	Assess long-term survival and functional integration of human iPSC-derived retinal transplants in primate models, examining light perception recovery	(Shirai et al., 2016; Tu et al., 2019; Yamasaki et al., 2021; Kajita et al., 2024)

## Data Availability

No data was used for the research described in the article.
